# Determinants of COVID-19 vaccination coverage in European and Organisation for Economic Co-operation and Development (OECD) countries

**DOI:** 10.3389/fpubh.2024.1466858

**Published:** 2024-12-31

**Authors:** Vladimira Varbanova, Niel Hens, Philippe Beutels

**Affiliations:** ^1^Centre for Health Economics Research and Modelling Infectious Diseases, Vaccine and Infectious Disease Institute, University of Antwerp, Antwerp, Belgium; ^2^Interuniversity Institute of Biostatistics and Statistical Bioinformatics (I-BioStat), Data Science Institute, Hasselt University, Hasselt, Belgium

**Keywords:** COVID-19, vaccination, determinants, OECD, European Union

## Abstract

**Introduction:**

In relatively wealthy countries, substantial between-country variability in COVID-19 vaccination coverage occurred. We aimed to identify influential national-level determinants of COVID-19 vaccine uptake at different COVID-19 pandemic stages in such countries.

**Methods:**

We considered over 50 macro-level demographic, healthcare resource, disease burden, political, socio-economic, labor, cultural, life-style indicators as explanatory factors and coverage with at least one dose by June 2021, completed initial vaccination protocols by December 2021, and booster doses by June 2022 as outcomes. Overall, we included 61 European or Organisation for Economic Co-operation and Development (OECD) countries. We performed 100 multiple imputations correcting for missing data and partial least squares regression for each imputed dataset. Regression estimates for the original covariates were pooled over the 100 results obtained for each outcome. Specific analyses focusing only on European Union (EU) or OECD countries were also conducted.

**Results:**

Higher stringency of countermeasures, and proportionately more older adults, female and urban area residents, were each strongly and consistently associated with higher vaccination rates. Surprisingly, socio-economic indicators such as gross domestic product (GDP), democracy, and education had limited explanatory power. Overall and in the OECD, greater perceived corruption related strongly to lower vaccine uptake. In the OECD, social media played a noticeable positive role. In the EU, right-wing government ideology exhibited a consistently negative association, while cultural differences had strong overall influence.

**Conclusion:**

Relationships between country-level factors and COVID-19 vaccination uptake depended on immunization stage and country reference group. Important determinants include stringency, population age, gender and urbanization, corruption, government ideology and cultural context.

## 1 Introduction

The COVID-19 pandemic had unprecedented effects worldwide: it disrupted industries bringing economies to the verge of recession, interrupted social life to a degree harmful to mental health, and put enormous strain on healthcare systems. By early May 2023, when the World Health Organization (WHO) declared the pandemic was over, 700 million confirmed cases and close to 7 million COVID-19 deaths had been reported (1), which is estimated at about a third of the actual COVID-19 death toll based on excess mortality data (2). Changes in demographics with life-expectancy decreasing after 2020 were observed in almost all European Union (EU) member states (3), as well as in many other places around the world (4). Governments implemented non-pharmaceutical interventions (NPI) to mitigate the COVID-19 disease burden and pressure on healthcare systems, but counted on vaccine development to build up immunity before these NPIs could be released. Thus, the rapid development of vaccines became a necessary, and, fortunately, very effective and cost-effective public health tool allowing for the gradual release of NPIs (5, 6). However, mass immunization progressed very unevenly around the world, arguably due to limited manufacturing capacity, global inequality in access and distribution, and vaccine demand and hesitancy (7). Among relatively wealthy countries where vaccine supply was generally similarly available to governments, vaccine uptake varied largely as well. Taking an exploratory approach, with the present study we aimed to investigate potential country-level determinants that might explain observed differences in national COVID-19 vaccination rates in relatively wealthy nations.

## 2 Materials and methods

### 2.1 Sample

Our selection of 61 countries included all European countries and Organisation for Economic Co-operation and Development (OECD) member states. We show country rankings according to gross national income (GNI) for 2019 in Appendix A. As per The World Bank (TWB)'s classification by income level (8) from the 1st of July 2020 (to be applied to 2019 GNI), all but one country fall either in the “high-income” or the “upper-middle income” brackets. In addition to performing the statistical analysis with the full sample of 61 countries, we also repeated it with less heterogeneous sub-samples of only EU member states (27 in total) and of only OECD countries (38 in total, 21 of which are also EU countries). The sub-sample of EU countries focuses not only on countries that are more alike economically (given EU regulations on international trade, budget deficit criteria, and the joint post-Brexit negotiation with the UK), but that also benefitted equally from large joint COVID-19 vaccine pre-orders of the EU commission on their behalf (9). The broader sub-sample of OECD countries does include non-EU European countries, as well as non-European countries. This allows for other determinants to dominate in a group that has other common traits than the EU subgroup, given that despite their diversity, OECD countries have all been historically accepted as members of the OECD for economic and geopolitical reasons. These sub-samples allow us to study how the selection of countries may influence the identification of influential determinants.

### 2.2 Outcome variables

**Figure 1:**
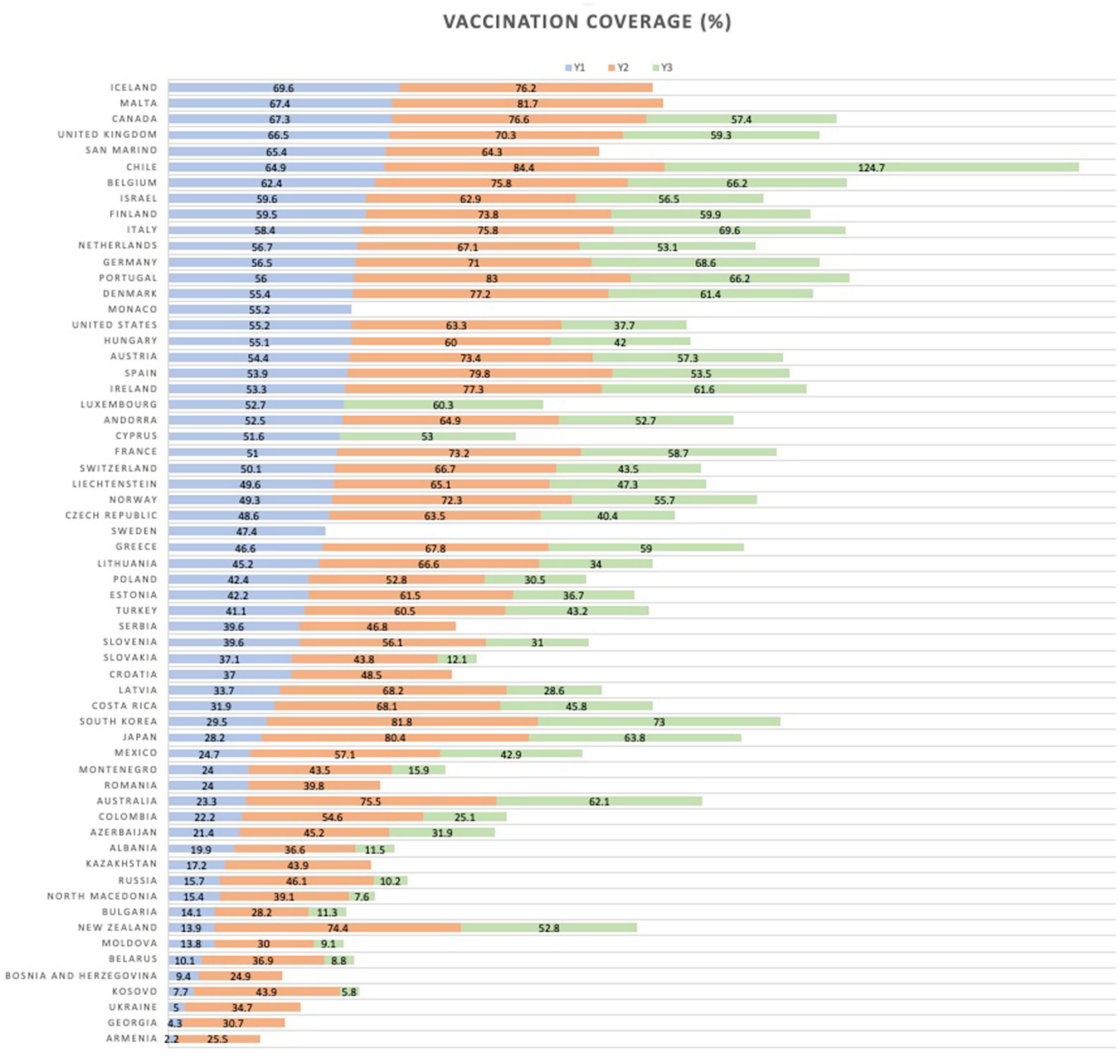
Vaccination coverage for the three outcomes.

Data on COVID-19 vaccination coverage was extracted from Our World in Data (OWiD)^1^ database on the 9th of February 2023. Specifically, based on key milestones in the progressive roll-out of the vaccine programs, we took the following variables' values 6 months apart, starting with the Y1 variable reflecting 1st dose coverage measured roughly 6 months after the start of the international immunization effort (see Figure 1).

Y1 - Total number of people who received at least one vaccine dose (per 100 people in the total population), as of the 30th of June 2021;

Y2 - Total number of people who received all doses prescribed by the initial vaccination protocol (per 100 people in the total population), as of the 31st of December 2021;

Y3 - Total number of COVID-19 vaccination booster doses administered (per 100 people in the total population), as of the 30th of June 2022;

In case of missing values for our particular time-points, we used linear interpolation between the two closest dates around the time-points with available data. The remaining missingness was 0% for Y1, 6.5% for Y2, and 21.3% for Y3.

### 2.3 Covariates

We considered a broad number of demographic, healthcare resource, disease burden, political, socio-economic, labor, cultural, life-style, and other factors in the current study. Table 1 lists all 53 covariates included. Data was collected from a number of well-known publicly accessible databases. Most data refers to 2019 (the last pre-pandemic year), with a few noted exceptions due to data inavailability (e.g., the democracy covariate was obtained from a project that ended in 2018) or for conceptual reasons (e.g., if available, we preferred more recent measurements of potentially volatile non-structural indicators, such as government orientation or social media audience). Six covariates reflecting COVID-19 disease burden, healthcare system pressure, and government response, varied in the covered time-period depending on outcome (see Table 1, rows 1, 2, 4–7), which resulted in three distinct cross-sectional datasets of 54 variables in total each-53 covariates and one outcome. Lastly, and as also noted in Table 1, four covariates were derived, composite variables, calculated from data provided by OWiD and the Oxford COVID-19 Government Response Tracker (OxCGRT) database (10). These four covariates are: intensive care unit (ICU) occupancy, average government response index (GRI) for non-vaccinated, average difference between GRI for non-vaccinated and vaccinated, and number of considered 6-month time-periods during which a requirement for mandatory vaccination was in place for at least one occupational group for at least 1 day.

**Table 1:** Covariates considered in the study.

	**Variable**	**Time period**	**Possible values**	**Missing data (%)**	**Source**	**Last accessed**
1	Total deaths attributed to COVID-19 per 1,000,000 people	30 Jun 21/31 Dec 21/30 Jun 22	Numeric	0	OWiD^a^	09.02.2023
2	Excess mortality (cumulative difference between the reported number of deaths since 1 January 2020 and the projected number of deaths for the same period based on previous years, per million people)	30 Jun 21/31 Dec 21/30 Jun 22	Numeric	3.3/3.3/9.8	OWiD^a^	09.02.2023
3	Hospital beds (per 1,000 people)	Most recent year available since 2010	Numeric	3.3	OWiD^a^	09.02.2023
4	ICU occupancy (proportion of time the ratio ICU patients/ICU beds > 0.60)^*^	1 Jan 20–30 Jun 21/1 Jan 20–31 Dec 21/1 Jan 20–30 Jun 22 (ICU beds data for 2020 or latest available)	Numeric (0 to 1)	57.4	OWiD^b^	25.02.2023
5	Average government response index (GRI) for non-vaccinated^*^	1 Jan 20–30 Jun 21/1 Jan 20–31 Dec 21/1 Jan 20–30 Jun 22	Numeric (0–100)	4.9	OxCGRT^c^	03.03.2023
6	Average difference between government response index (GRI) for non-vaccinated and vaccinated^*^	1 Jan 21–30 Jun 21/1 Jan 21–31 Dec 21/1 Jan 21–30 Jun 22	Numeric (0–100)	4.9	OxCGRT^c^	03.03.2023
7	Mandatory vaccination (duration of a policy requirement to be vaccinated in order to work in a specific occupation, or for a specific group to be vaccinated)^*^	1 Jan 21–30 Jun 21/1 Jan 21–31 Dec 21/1 Jan 21–30 Jun 22	Numeric (0/1; 0/1/2; 0/1/2/3)	4.9	OxCGRT^c^	25.02.2023
8	Facebook audience (as % of the population aged 13+)	2021	Numeric (0–100)	0	DataReportal^d^	17.02.2023
9	Twitter audience (as % of the population aged 13+)	2021	Numeric (0–100)	0	DataReportal^d^	17.02.2023
10	Birth rate, crude (per 1,000 people)	2019	Numeric	1.6	TWB^e^	09.02.2023
11	Current health expenditure (% of GDP)	2019	Numeric (0–100)	4.9	TWB^e^	09.02.2023
12	GDP per capita, PPP (current international $)	2019	Numeric	4.9	TWB^e^	09.02.2023
13	DPT immunization (% of children ages 12–23 months)	2019	Numeric (0–100)	3.3	TWB^e^	09.02.2023
14	Measles immunization (% of children ages 12-23 months)	2019	Numeric (0–100)	3.3	TWB^e^	09.02.2023
15	Labor force, female (% total labor force)	2019	Numeric (0–100)	8.2	TWB^e^	09.02.2023
16	Population ages 15–64 (% of total)	2019	Numeric (0–100)	0	TWB^e^	09.02.2023
17	Population ages 65 and above (% of total)	2019	Numeric (0–100)	0	TWB^e^	09.02.2023
18	Population density (people per sq. km of land area)	2019	Numeric	1.6	TWB^e^	09.02.2023
19	Population growth (annual %)	2019	Numeric (0–100)	0	TWB^e^	09.02.2023
20	Population, female (% of total population)	2019	Numeric (0–100)	0	TWB^e^	09.02.2023
21	Population, total	2019	Numeric	0	TWB^e^	09.02.2023
22	Poverty headcount ratio at national poverty lines (% of population)	2019	Numeric (0–100)	34.4	TWB^e^	09.02.2023
23	Research and development (R&D; gross domestic expenditures, % of GDP)	2019	numeric (0 to 100)	11.5	TWB^e^	09.02.2023
24	Surface area (sq. km)	2019	Numeric	1.6	TWB^e^	09.02.2023
25	Unemployment, total (% of total labor force; modeled ILO estimate)	2019	Numeric (0–100)	8.2	TWB^e^	09.02.2023
26	Urban population (% of total)	2019	Numeric (0–100)	1.6	TWB^e^	09.02.2023
27	Political stability and absence of violence/terrorism	2019	Numeric (−2.5 to 2.5)	0	TWB^f^	08.02.2023
28	Democracy (Polity2 index: revised combined Polity score = Institutionalized Democracy score – Institutionalized Autocracy score)	2018	Numeric (−10 to 10)	11.5	Center for Systemic Peace^g^	16.02.2023
29	Government composition: % cabinet posts of right-wings parties	2020	Numeric (0–100)	41.0	Comparative Political Data Set 1960–2020^h^	15.02.2023
30	Economic freedom (the degree to which the policies and institutions of countries are supportive of economic freedom)	2019	Numeric (0–10)	8.2	Fraser Institute^i^	08.02.2023
31	Gini index of income inequality	2019	Numeric (0–100)	0	UN University UNU-WIDER^j^	14.02.2023
32	Education (average number of completed years of education of a country's population aged 25 years and older, excluding years spent repeating individual grades)	2019	Numeric	63.9	UNESCO^k^	14.02.2023
33	Disability-adjusted life years (DALYs; per 100,000 people)	2019	Numeric	3.3	Institute for Health Metrics and Evaluation^l^	14.02.2023
34	Corruption perception index (CPI)	2019	Numeric (0 to 100)	6.5	Transparency International^m^	14.02.2023
35	Alcohol consumption (in liters of pure alcohol; total per capita, age 15+)	2019	Numeric	6.5	WHO^n^	14.02.2023
36	Nurses and midwives (per 10,000 people)	2019	Numeric	42.6	WHO^n^	14.02.2023
37	Medical doctors (per 10,000 people)	2019	Numeric	32.8	WHO^n^	14.02.2023
38	Pharmacists (per 10,000 people)	2019	numeric	45.9	WHO^n^	14.02.2023
39	Regular daily smokers (% of in the population, age 15+)	2019	Numeric (0–100)	42.6	WHO^#o;^ OECD^p^	13.02.2023
40	Social expenditure (% of GDP; total for the main social policy areas: old age, survivors, Incapacity-related benefits, family, active labor market programmes, unemployment, housing, and other social policy areas)	2019	Numeric (0–100)	37.3	OECD^p^	16.02.2023
41	Total health care coverage; government/social health insurance (% of population)	2019	Numeric (0–100)	39.3	OECD^p^	13.02.2023
42	Total health and social employment density (per 1,000 people)	2019	Numeric	39.3	OECD^p^	13.02.2023
43	Psychiatrists density (per 1,000 people)	2019	Numeric	45.9	OECD^p^	13.02.2023
44	Surgical group of specialists density (per 1,000 people)	2019	Numeric	45.9	OECD^p^	13.02.2023
45	Total hospital employment density (per 1,000 people)	2019	Numeric	55.7	OECD^p^	13.02.2023
46	Hospitals (per 1,000,000 people)	2019	Numeric	42.6	OECD^p^	13.02.2023
47	Long-term care (LTC) recipients in institutions (other than hospitals; % of total population aged 65+)	Time-invariant	Numeric (0–100)	55.7	OECD^p^	20.02.2023
48	Power distance (“the degree to which the less powerful members of a society accept and expect that power is distributed unequally”)	Time-invariant	Numeric (0–100)	29.5	Hofstede 6-D model of national culture^q^	15.02.2023
49	Individualism (v collectivism; “a preference for a loosely-knit social framework in which individuals are expected to take care of only themselves and their immediate families”)	Time-invariant	Numeric (0–100)	29.5	Hofstede 6-D model of national culture^q^	15.02.2023
50	Masculinity (v femininity; “a preference in society for achievement, heroism, assertiveness, and material rewards for success”)	Time-invariant	Numeric (0–100)	29.5	Hofstede 6-D model of national culture^q^	15.02.2023
51	Uncertainty avoidance (“the degree to which the members of a society feel uncomfortable with uncertainty and ambiguity”)	Time-invariant	Numeric (0–100)	29.5	Hofstede 6-D model of national culture^q^	15.02.2023
52	Long-term orientation (v short-term orientation; the degree to which society holds the notion that the world “is in flux, and preparing for the future is always needed” as opposed to the view that “the world is essentially as it was created, so that the past provides a moral compass”)	Time-invariant	Numeric (0–100)	13.1	Hofstede 6-D model of national culture^q^	15.02.2023
53	Indulgence (v restraint; the extent to which society “allows relatively free gratification of basic and natural human drives related to enjoying life and having fun”)	Time-invariant	Numeric (0–100)	13.1	Hofstede 6-D model of national culture^q^	15.02.2023

^*^Composite variable derived from own calculations (see Section 2.3).

^#^Primary source.

OWiD, Our World in Data; OxCGRT, Oxford COVID-19 Government Response Tracker; TWB, The World Bank; UN, United Nations; UNESCO, United Nations Educational, Scientific and Cultural Organization; OECD, Organisation for Economic Co-operation and Development; WHO, World Health Organization.

^a^ https://github.com/owid/covid-19-data/tree/master/public/data

^b^ https://ourworldindata.org/covid-hospitalizations

^c^ https://github.com/OxCGRT/covid-policy-tracker

^d^ https://datareportal.com/library

^e^ https://databank.worldbank.org/source/world-development-indicators

^f^ http://info.worldbank.org/governance/wgi/

^g^ https://systemicpeace.org/inscr/p5v2018.xls

^h^ https://www.cpds-data.org/index.php/data

^i^ https://www.fraserinstitute.org/economic-freedom/dataset?geozone=world&page=dataset&min-year=1970&max-year=2018&filter=1&date-type=range

^j^ https://www.wider.unu.edu/database/world-income-inequality-database-wiid

^k^ http://data.uis.unesco.org/#

^l^ https://vizhub.healthdata.org/gbd-results/

^m^ https://www.transparency.org/en/cpi/2019

^n^ https://apps.who.int/gho/data/view.main.UHCHRHv

^o^ https://gateway.euro.who.int/en/datasets/european-health-for-all-database/

^p^ https://stats.oecd.org/#

^q^ https://geerthofstede.com/research-and-vsm/dimension-data-matrix/

### 2.4 Statistical analysis

Like many real-life datasets, ours, too, were characterized by some data missingness and multicollinearity, which limit the applicability of simple statistical approaches. Overall missingness varied between 18 and 18.6% in the three datasets (information per variable is presented in Table 1). For the covariate cumulative excess mortality, we collected data for the exact same dates as the outcomes. However, statistics for some countries were reported only weekly, so whenever necessary we applied linear interpolation, just like for our outcomes, in order to obtain estimates for the precise dates that we needed. In order to deal with the remaining missingness for this and all other variables, we performed multiple imputation by chained equations (MICE) in R (11). Multiple imputation (MI) is the current “state-of-the-art” approach to handling missing data, as it accounts for the inherent uncertainty when imputing, that gets ignored by single imputation techniques. Under the missing-at-random (MAR) assumption, we implemented the MICE algorithm, also known as fully conditional specification (FCS). MICE does not assume a multivariate distribution for the data, but instead uses a set of conditional densities. Imputation is done on a variable-by-variable basis, iterating over a conditionally specified imputation model for each incomplete variable. We used the predictive mean matching (PMM) method, which entails first calculating predictions for each entry of the target variable, whether observed or missing. For each missing entry then, a small number of “candidate donors” among the observed cases is selected, based on proximity between the predictions. At the end, one donor from the group is randomly selected and its observed value is imputed for the missing entry. Main advantages of this method include: it ensures that the imputed value is always within the plausible range as it is based on observed data; it has the ability to handle all types of variables (our datasets contained both continuous and categorical variables); and it is robust to transformations. In order to facilitate the MI procedure, we standardized all continuous variables to have a mean of 0 and a standard deviation of 1. We obtained 100 imputed versions of each of the three separate incomplete datasets.

Subsequently, we employed partial least squares regression (PLSR) (12) with each of these imputed datasets individually, using SAS. With an origin in chemometrics, PLSR has become one of the most widely used multivariate dimension-reduction techniques across disciplines. It combines projection and multiple regression into one, with the advantage of being able to handle a large (relative to sample size) number of both continuous and categorical variables exhibiting multicollinearity, which was, indeed, a feature of our datasets. The core assumption of PLSR is that the process under study is, in fact, influenced by just a few underlying latent variables (13) (also sometimes called factors or components), which constitute orthogonal linear combinations of the original variables, created in a way as to identify the directions within the predictor-space (the X-space) that explain as much as possible of the variance in the response-space (the Y-space) (14). The procedure also produces regression estimates for the original predictors, or covariates. In order to identify the country-level indicators most strongly associated with COVID-19 vaccination rates, we averaged the PLSR coefficients for each covariate over the 100 imputations for each of our three datasets separately.

## 3 Results

Table 2 presents the pooled results for our three outcome variables (proportion of the population with at least one dose, proportion fully vaccinated, and booster uptake) for the full sample of 61 countries, the EU sub-sample, and the OECD sub-sample. In the table, we have used red coloring to indicate a positive relationship and blue to indicate a negative one, with the intensity of the color reflecting the relative magnitude of the effects. Additional statistics around the mean effects shown here are presented in Appendix B, according to outcome and country sample. As a general rule-of-thumb, effects of more than 0.05 in absolute value can be considered statistically significant based on 95% confidence intervals (CIs) as reported in Appendix B.

**Table 2:** Mean partial least squares regression (PLSR) effects in the full sample of 61 European and OECD countries, in EU countries only, and in OECD countries only, over 100 imputed datasets for each outcome (color intensity reflects effect strength, red for positive and blue for negative direction of the relationship).

	**All 61 countries**			**EU**			**OECD**		
	**Y1**	**Y2**	**Y3**	**Y1**	**Y2**	**Y3**	**Y1**	**Y2**	**Y3**
Alcohol consumption	−0.044	0.053	−0.211	0.027	0.091	−0.020	−0.073	0.138	0.085
Area	0.045	−0.068	−0.160	−0.051	−0.008	0.027	0.120	−0.026	−0.189
Birth rate	−0.053	−0.289	−0.098	−0.137	−0.056	0.049	−0.176	−0.281	−0.379
Corruption perception^*^	0.240	0.116	0.415	0.070	0.023	−0.038	0.320	0.095	0.313
DALYs	−0.188	−0.152	−0.157	−0.112	−0.090	−0.029	−0.008	−0.175	−0.140
Democracy	−0.166	−0.049	0.036	0.076	−0.093	−0.021	0.022	−0.022	−0.031
Difference in GRI	−0.034	0.034	0.000	−0.042	−0.020	−0.044	−0.047	0.084	0.066
DPT immunization	−0.032	0.085	−0.063	−0.018	0.026	0.002	−0.351	−0.093	−0.098
Economic freedom	−0.200	0.112	0.058	0.097	0.204	0.070	−0.262	0.247	−0.079
Education	−0.007	−0.012	−0.093	−0.045	−0.012	−0.141	−0.027	−0.005	−0.176
Excess mortality	0.004	−0.197	−0.258	−0.094	−0.110	−0.194	−0.004	−0.057	−0.345
Facebook audience	0.169	0.063	−0.098	0.126	0.068	0.081	0.395	0.323	0.146
Female labor	−0.102	−0.116	−0.179	0.010	0.093	−0.088	−0.085	−0.220	−0.203
Female pop	0.235	0.320	0.320	0.102	0.246	0.080	0.260	0.345	0.138
GDP	0.054	−0.037	−0.158	0.000	−0.010	0.105	0.001	0.060	−0.210
Gini	−0.081	0.051	0.203	−0.189	−0.073	0.002	−0.105	0.072	0.469
GRInon-vaccinated	0.403	0.185	0.300	0.075	0.209	0.112	0.403	0.089	0.271
Health and social employment	0.051	−0.056	0.220	−0.014	−0.053	0.108	0.077	−0.028	0.337
Health expenditure	−0.105	−0.071	0.017	0.020	0.078	−0.021	−0.098	0.139	0.018
Hospital beds	−0.386	−0.081	0.127	−0.011	0.009	0.011	−0.156	0.053	0.213
Hospital density	−0.097	0.028	−0.143	−0.018	−0.017	−0.048	−0.174	−0.010	−0.216
Hospital employment	−0.010	−0.050	−0.109	−0.024	0.005	0.061	0.056	−0.030	−0.113
ICU occupancy	−0.048	0.012	−0.047	0.049	0.000	0.083	−0.075	−0.013	−0.008
Individualism	0.049	−0.003	−0.011	0.192	0.159	−0.009	−0.091	−0.114	−0.087
Indulgence	−0.108	0.049	0.106	0.014	−0.044	0.012	−0.394	−0.082	−0.171
Long-term care residents	0.048	0.028	−0.058	0.043	−0.050	−0.059	−0.074	−0.084	−0.084
Long-term orientation	−0.008	−0.064	−0.166	0.010	−0.070	−0.054	−0.056	−0.150	−0.240
Mandatory vaccination	−0.449	0.092	0.053	0.012	0.040	0.032	−0.387	0.030	−0.003
Masculinity	−0.030	−0.089	−0.155	0.148	0.012	0.092	−0.008	−0.060	−0.047
MCV1 immunization	−0.029	−0.083	0.011	0.002	0.053	−0.088	0.246	0.166	−0.205
MDs density	−0.118	−0.054	0.037	−0.012	−0.038	−0.003	−0.064	−0.046	−0.006
Nurses and midwives density	−0.029	0.076	0.119	0.000	0.039	0.129	−0.033	0.094	0.143
Pharmacists density	0.022	0.075	0.133	−0.008	0.040	0.038	0.042	0.109	0.231
Political stability and lack of violence/terrorism	0.183	0.051	−0.182	0.055	0.015	−0.007	−0.146	−0.126	−0.289
Population 15–64 yrs	0.106	0.021	0.118	0.040	−0.039	−0.081	0.169	0.100	0.062
Population 65+	0.481	0.121	0.140	0.081	0.047	0.001	0.156	0.064	0.117
Population density	0.053	−0.137	−0.071	−0.006	0.033	−0.015	−0.237	−0.200	0.068
Population growth	0.133	0.167	−0.054	0.019	0.105	−0.213	0.059	−0.114	−0.075
Population size	−0.023	0.106	−0.218	−0.002	−0.059	0.065	−0.092	−0.034	−0.268
Poverty	−0.020	−0.064	−0.122	−0.061	0.000	0.040	−0.047	−0.050	−0.188
Power distance	−0.082	−0.094	0.074	−0.102	−0.200	−0.126	−0.130	−0.106	0.006
Psychiatrists density	0.087	−0.042	−0.009	−0.008	−0.072	0.023	0.311	−0.049	0.052
R&D expenditure	0.226	0.073	−0.023	0.033	0.001	0.119	0.278	0.067	0.014
Right-wing in government	−0.032	−0.031	−0.063	−0.118	−0.139	−0.114	−0.074	0.000	0.006
Smoking prevalence	0.024	−0.025	−0.019	−0.037	−0.052	−0.050	−0.025	−0.037	−0.089
Social expenditure	0.002	−0.020	−0.152	0.078	0.076	0.037	0.006	−0.046	−0.188
Surgeons density	0.067	0.069	0.129	0.026	0.056	0.079	−0.005	0.028	0.113
Total deaths	0.121	−0.136	0.158	0.012	−0.092	−0.052	0.333	−0.090	0.160
Total healthcare coverage	−0.161	0.032	−0.163	0.005	0.051	0.016	−0.071	0.153	−0.123
Twitter audience	0.042	0.049	0.111	0.032	0.052	0.026	0.276	0.129	0.043
Uncertainty avoidance	0.099	0.122	0.095	0.169	0.170	0.167	−0.040	0.078	−0.053
Unemployment rate	−0.168	−0.128	−0.147	−0.022	0.037	0.008	−0.125	−0.079	−0.185
Urban population	0.114	0.106	0.137	0.130	0.119	0.163	0.316	0.258	0.398

^*^The corruption perception index (CPI) scale runs from 0 = “highly corrupt” to 100 = “very clean”.

In the full sample of 61 European or OECD countries, with regards to Y1, or the vaccination rate with at least one dose, the covariates with the strongest positive relationship were population 65+ years of age (0.48), GRI for non-vaccinated (0.40), corruption perception (to be noted that the scale runs from 0 = “highly corrupt” to 100 = “very clean”; coefficient 0.24), female proportion of population (0.23), and research and development (R&D) expenditure (0.23). By contrast, mandatory vaccination (−0.45), hospital beds (−0.39), and economic freedom (−0.20) showed the strongest negative relation. With regards to Y2, or the proportion of people who completed the initial vaccination protocol, associations were overall weaker. Female population showed the strongest positive effect by a long shot (0.32), while birth rate exhibited a relatively strong negative effect (−0.29). With regards to Y3, or booster vaccination rate, less perceived corruption showed again a very strong positive relationship (coefficient 0.41), followed by, again, female population (0.32), GRI for non-vaccinated (0.30), health and social employment (0.22), and the Gini coefficient of inequality (0.20). The strongest negative associations were found with excess mortality (−0.26), population size (−0.22), and alcohol consumption (−0.21).

Looking at the results from the EU sub-sample, we observe somewhat weaker effects overall, when compared to those from the full sample. The top 3 covariates positively associated with Y1 were the culture indicators individualism (0.19), uncertainty avoidance (0.17), and masculinity (0.15); with respect to Y2, these were female proportion of population (0.25), GRI for non-vaccinated (0.21), and economic freedom (0.20); and with respect to Y3—uncertainty avoidance (0.17), proportion of urban population (0.16), and nurses and midwives density (0.13). On the other hand, the Gini inequality coefficient (−0.19), birth rate (−0.14), and right-wing government (−0.12) for Y1, power distance (−0.20), right-wing government (−0.14), and excess mortality (−0.11) for Y2, and population growth (−0.21), excess mortality (−0.19), and average education (−0.14) for Y3 were the factors with the most pronounced negative association observed within the EU.

Lastly, in the sub-sample of OECD countries, we see many larger effects, both positive and negative. GRI for non-vaccinated (0.40), Facebook audience (0.39), total deaths (0.33), corruption perception (0.32; reminder that scale goes from 0 = “highly corrupt” to 100 = “very clean”), urban population (0.32), and psychiatrists density (0.31), followed by a few others, exhibited noteworthy positive relationship with Y1, while indulgent culture (−0.39), mandatory vaccination (−0.39), and diphtheria toxoid, pertussis, and tetanus toxoid (DPT) immunization (−0.35) were the indicators with the strongest negative relation. With regards to Y2, female population (0.34), Facebook audience (0.32), urban population (0.26), and economic freedom (0.25) showed the strongest positive effects, while birth rate (−0.28), female labor (−0.22), and population density (−0.20) were the top 3 negatively associated factors. Variations in Y3 in the OECD context were best explained by its positive relations with the Gini (0.47), proportion of urban population (0.40), health and social employment (0.34), corruption perception (0.31; to be interpreted inversely, as the scale runs from 0 = “highly corrupt” to 100 = “very clean”), GRI for non-vaccinated (0.27), pharmacists density (0.23), and hospital beds (0.21), as well as its negative relations with birth rate (−0.38), excess mortality (−0.34), political stability and lack of violence/terrorism (−0.29), population size (−0.27), long-term oriented culture (−0.24), hospital density (−0.22), gross domestic product (GDP; coeficient of −0.21), immunization with the first dose of measles-containing vaccines (MCV1; estimate of −0.20), and female labor participation (−0.20).

## 4 Discussion

Using a data-driven approach, with the present study we aimed to identify the most important country-level determinants explaining variations in COVID-19 vaccine uptake among relatively wealthy countries. Scarce research to-date (and referenced below) has generally used a larger and more contextually diverse mix of countries while focusing on a relatively small number of determinants. Our results paint a very dynamic picture, both in terms of magnitude of the covariates' influence as well as direction of relationships. Based on the obtained 53 covariates' MI-PLSR estimates, we can say that the driving forces behind vaccination success depended on: (1) the phase of the immunization effort, the progression of which we tried to capture by using these specific outcome variables at those specific points in time; and (2) the sample of countries considered—despite a huge overlap between our two sub-samples of EU and OECD countries, the results were markedly different, with many more pronounced effects in the latter. Nevertheless, a number of determinants can be acknowledged as indisputably important, and certain patterns can be discerned.

First and foremost, the overall strongest effect observed in our analysis was the positive one of proportion of the population 65+ years of age for Y1 in the full sample. As the older adults were recognized as the most at-risk group and given priority for vaccination against COVID-19, it makes sense that their proportion in a nation's population was one of the propelling forces at the start of the vaccination campaign. However, this was only visible in the full, most diverse, sample of countries, while in the OECD context and especially in the EU the effect was negligible. To our knowledge, there are just two other studies, one with a sample of 89 countries (73) and another investigating vaccination at the state level in the US (16), that found a similar positive effect for that covariate.

Next, we want to draw attention to the three covariates of which the effects remained significant (in terms of size) and positive over time and across samples. Proportion of females in the population turned out to be consistently influential in the full sample of 61 countries, exhibiting the largest effect for Y1 in the OECD, and showing a more variable impact in the EU sub-sample, but still with one of the strongest effects for Y2. Individual-level research provides a few plausible explanations for the positive relationship observed here. To start with, females are known to utilize preventive care more often than males (17). They also have higher general (18) as well as COVID-specific risk perception (19, 20), and tend to see COVID-19 as a serious health problem to a larger extent than their male counterparts (21). Starting early on in the pandemic, research conducted in EU and OECD countries established that women hold more positive views of and exhibit greater compliance with NPIs such as social distancing, face-covering, and hygiene (19–27). It has been hypothesized that this relationship between gender and compliance is moderated by conscientiousness and agreeableness (24)—two of the Big Five personality traits that have already been connected to compliance in their own right (20, 28–32).

The second factor with consistent beneficial effect on COVID-19 vaccine uptake in our analysis was proportion of the population living in urban areas. While some previous studies have failed to find a relationship (33, 34), our results showed that urbanicity was one of the strongest and most stable influences. A possible explanation for this could be that the perception of crowdedness in urban settings promotes higher COVID-19 risk-perception and consequent willingness to vaccinate against it. In addition, vaccination logistics are easier to manage in more urbanized countries, irrespective of how vaccines were predominantly delivered—e.g., at large vaccination centers, general practitioner (GPs) offices, pharmacies, via mobile outreach, etc. This seems especially relevant later in the vaccination campaign, with the booster dose (our Y3), when we can assume general risk-perception and urgency to vaccinate may have subsided.

The third consistently positive and very important determinant we discovered was the GRI—a summary measure, based on policies and restrictions for non-vaccinated individuals in a country. We note that a related composite variable we used, expressing the difference in GRI between vaccinated and non-vaccinated persons, did not emerge as being important. That is, while restricting the liberties of people was influential, the extent to which these restrictions depended on vaccination status alone was not. The GRI effect was especially pronounced in the initial stage of vaccination in the full sample and the OECD sub-sample, while its strongest influence in the EU was observed at completing the initial vaccination protocol (or Y2). Extant literature has studied the relationship between COVID-19 vaccination coverage and stringency (35, 73) and vaccine passes (36) to mixed results. Based on our findings, however, we conclude that restrictive policies were, indeed, effective in incentivizing people to vaccinate. In connection to that, the strongest negative association for Y1 in the full and OECD samples was exhibited by our mandatory vaccination variable—another composite index reflecting whether and for how many 6-month periods, as defined by our outcome time-points, a policy requirement for mandatory vaccination for at least one occupational group was in place. The negative direction of this nexus can be explained by reverse causality, as such requirements were typically mandated as a response to low vaccine uptake during the initial phase of the vaccination campaign. Indeed, later on the effect of this covariate lessened in magnitude significantly and changed signs to positive, or just disappeared (in the EU context, it was always non-existent). Such reverse causality is also suspected with regards to the excess mortality determinant, whose negative relation with vaccination coverage increased in all three country groups as vaccination efforts progressed (i.e., the negative association became stronger moving from Y1 to Y2 to Y3).

As a reaction to government imposed NPIs and mandates for mandatory vaccination, there were numerous voices expressing grievances that these measures were in breach of civil liberties, which are considered “a hallmark feature of liberal democracies” (35). There have also been suggestions that countries with authoritarian political regimes are at an advantage in handling the pandemic, as their governments could act more decisively without fearing electoral consequences (37, 38). Empirical support for this statement can be potentially found in studies regarding EU and high-income countries, where higher democracy levels were linked to more COVID-related deaths early in the pandemic (39, 40). However, when the relationship with vaccination coverage is tested, previous research shows conflicting results with regards to civil liberties (35, 73) and no effect of democracy itself (33, 34). Our results using the Polity2 index to capture democracy levels seem to largely agree: in our sample of relatively wealthy countries, democracy had a somewhat small negative effect on Y1 in the full sample, negligible and inconsistent in terms of direction association in the EU sub-sample, and practically no influence in the OECD.

Among the other political climate factors we included in the analysis, proportion of right-wing politicians in the government exhibited small to non-existent effects in the full sample of 61 countries and the OECD sub-sample, but consistent negative influence of moderate strength, relatively speaking, in the EU context. We cannot speculate whether governments with higher number of right-wing members are just less efficient, or, perhaps, less able or willing to organize and execute a successful vaccination campaign in the middle of a pandemic. However, this finding seems to be complementary to results from another EU study where average political orientation of the population to the right was associated with greater resistance to COVID-19 vaccination (41). Furthermore, the variable of political stability and absence of violence/terrorism showed an effect swinging from positive at Y1 to the exact same negative equivalent at Y3 in the full sample, very small influence in the EU, and a consistently negative, at Y3 even noticeably strong, effect in OECD countries. A number of previous studies attempting to investigate the relationship between political stability and COVID-19 vaccination coverage around the world have discovered a positive connection (34, 42–45), which only makes sense, given that “the political stability of a country is crucial for regulating the vaccination campaign, communicating the content congruent with government action to citizens, and effectively organizing mass vaccination” (45). Still, what we observed in our results could perhaps be explained by the fact that our sample consists of predominantly strong democracies that are characterized by stability and lack of violence.

One of the most interesting and important factors to discuss in the context of COVID-19 vaccination and, in fact, the whole pandemic, is corruption. Healthcare is considered a sector particularly susceptible to corrupt practices (46) and corruption in general has been shown to have detrimental effects on population health (47, 48). Channels via which corruption could affect the vaccination process have been identified in the literature (37), but there is somewhat mixed empirical evidence of the possible impact corruption perception and corruption control might have had on vaccine uptake (15, 33, 34, 42). In the present study, we used the Transparency International's Corruption perception index (CPI)—a principal corruption indicator covering corruption manifestations such as bribery, nepotism, and diversion of public funds (49). In our results, the effect is displayed as positive; however, it must be kept in mind that the index scale goes from 0 = “highly corrupt” to 100 = “very clean”. Thus, we see that while corruption perception was not an important factor for EU-only vaccination, in our full sample as well as in the OECD sub-sample, and for Y1 and Y3 in particular, countries perceived by their citizens as “clean” to a greater degree had higher numbers of vaccinated people, all else held constant. We endorse the assertion (33) that at least part of that effect is mediated via social and political trust, shown to be eroded by corruption (50, 51). We can thus conjecture, that these factors were especially pertinent at the beginning of the vaccination process (at Y1), when uncertainy about the so-quickly developed vaccines was high, as well as at the time the booster dose was being administered (at Y3). By Y3, steep pandemic waves of severe disease and death had largely disappeared and the urgency for vaccination had declined, leaving room for attitudes such as trust in the government, science, or, indeed, conspiracy theories [all of which have been linked to willingness to vaccinate (41), and for which we could not account directly] to play a bigger role. In addition, we must aknowledge that corruption is a known threat to data integrity, and intentional underreporting of COVID-related indicators is almost certain to have occurred in some places (43).

In addition to the older adult and female population, the role of other demographic covariates needs some reflection. While relatively strong associations were spotted sporadically, it is overall hard to identify any of these factors as categorically significant drivers of COVID-19 vaccine uptake, especially since depending on the phase of the vaccination effort and sample of countries, these associations changed sign as well. Population size showed a relatively big negative effect for the booster dose, Y3, in the full sample and OECD sub-sample [to mixed previous evidence (34, 35, 42, 45)]. Population growth had a remarkable negative effect for Y3 in the EU—in fact, the largest effect for the EU sub-sample regardless of outcome. Population growth can be viewed as the combined effect of the difference between births and deaths, and that between emigration and immigration, the latter stimulating population growth directly and also indirectly, with their offspring. There is evidence in the literature that, indeed, immigrant groups in the EU have lower routine vaccination coverage (52) as well as lower COVID-19 vaccine uptake (53–55). Furthermore in this “demographics” group of covariates, population density was a relatively strong negative influence for Y1 and Y2 in OECD countries (while it was found non-significant in previous studies on COVID-19 vaccination (16, 34, 35, 45), proportion of 15–64 year-olds exhibited a small positive relation with all outcome variables in the full sample and OECD sub-sample, and birth rate had negative associations in all samples, but growing in magnitude over time to notable levels in the OECD at Y3.

We can also make some interesting observations regarding socio-economic determinants. At the start of vaccination (Y1), economic freedom had a negative relation that changed to positive at the time of full vaccination protocol (Y2) in the full and OECD-only samples, while this covariate had always a positive effect in the EU, strongest at Y2. Female labor participation and unemployment rate exhibited similar patterns, where they had negative associations of moderate strength with almost all outcomes in the full and OECD samples [confirming previous evidence (56)], while practically very little to no influence in the EU context. GDP per capita, a factor previously recognized as crucial for general population health (57, 58), routine childhood (59) and COVID-19 vaccination uptake (42, 45, 60, 73), showed to be virtually immaterial for explaining between country differences in our analysis (except, perhaps, at Y3 in the full and OECD samples, where we observed a negative relation of about moderate strength). On the other hand, again, for the full and OECD samples, the Gini coefficient started as a slightly negative influence at Y1, but turned into one of the important positive factors by Y3; in fact, the most important for the OECD, with an effect size surpassing all others but one when looking at all samples and outcomes. A previous study finding a similar positive effect of the Gini coefficient argued that the free availability of the vaccines promoted uptake in the countries high on income inequality (45). This hypothesis was not supported by our EU-only results where we observed a very different dynamic: the Gini started at Y1 as a very strong negative effect, but quickly diminished to non-existent. This means that countries within the EU with higher levels of income inequality had a less successful, or perhaps slower, start of the vaccination campaign, but quickly managed to catch up. A conceptually connected covariate, poverty was overall not important [as in previous research (16)], until Y3 in the full and OECD samples when it showed a somewhat small negative association. Lastly here, we want to mention education. While other studies have discovered a positive relation (15, 73), our investigation yielded only non-significant or relatively small to moderate negative associations (mostly for the booster dose, Y3, and in the separate EU and OECD sub-samples) between average years of schooling and vaccine uptake. We speculate that the nexus between education and COVID-19 immunization rates may also depend on economic development, as these other studies did not focus on wealthy nations like we did. Our results of this slightly negative impact of education on COVID-19 vaccination find some support in evidence from previous EU research on the individual level as well (41). This slight negative effect, in particular on the booster dose, may also be explained by younger and higher educated populations on average feeling less inclined to take the booster dose, also in part because many had incurred SARS-CoV-2 infection in the period between Y2 and Y3, and in that case the marginal benefits of a booster dose were negligible. Furthermore, the Y3 booster dose was recommended most strongly for the older and frailer/unhealthier of any age, traits which are often found to be associated with lower education in individual level analyses.

Throughout the pandemic, there has been a “mountain” of both qualitative and quantitative research using social media data for the purpose of answering all sorts of questions, many of which connected to attitudes toward the virus and government measures, the spread of misinformation, and social media's role in the “infodemic”. In the context of our study, we used national audience on Facebook and Twitter measured by number of accounts divided by the total population. Of course, these were imperfect variables, as accounts on these platforms do not necessarily belong to an individual, one individual can have multiple accounts, accounts of deceased people remain, and so on. Still, we found them acceptable proxies for levels of connectedness and freedom of speech in society, which is a positive interpretation. This view on social media involvement was supported by our results, even if effect sizes were notably large only at certain times in certain samples of countries. Precisely, Twitter involvement was not a significant factor in the EU, or even in the full sample of 61 European and OECD countries, but it exhibited a relatively strong positive association with vaccination at the initial stage of the campaign in OECD countries only. Of the two, Facebook was the social media with more significant impact overall, and, again, this was most pronounced in the OECD sub-sample, where for both Y1 and Y2, or roughly the first year after the introduction of COVID-19 vaccines, Facebook audience was the second strongest positive factor (second strongest overall as well, taking negative associations into account, too).

With regards to the broad category of health-related covariates, we need to state right up front that those had minimal significance in the EU overall; so unless otherwise specified, the reader is to assume the patterns and relationships discussed here were found in the full and OECD samples. First, we focus on number of hospital beds relative to population size. This determinant had a particularly strong negative effect at Y1 in the full sample, which, we hypothesize, may have been mediated by (perception of) healthcare system pressure—the more hospital beds available, the greater the theoretical ability of the system to handle a given COVID-19 burden (61). Therefore, residents may feel greater need for immunization in order to minimize the burden on more pressurized systems, on the one hand, and to minimize their own risk of needing to rely for care on an overburdened system, on the other hand. By Y3 though, the relation between hospital beds and vaccination turned positive. At this later point in the pandemic, this variable could also be seen as a proxy for healthcare system organization—a possible explanation for the positive effect. Hospital density though, just like total healthcare coverage, exhibited negative association for Y1 and Y3 in particular. Somewhat surprising, proportion of the time (in the respective time-periods) that ICU occupancy by COVID-19 patients was 60% or more was largely ignorable as a possible reason for between country differences in vaccine uptake. We would presume this determinant would also act via perception of disease burden, just like total deaths as well—a factor with, indeed, positive connection with vaccination rates at Y1 and Y3, but a perplexing (smaller) negative one for Y2—the outcome in-between. Disability-adjusted life years (DALYs), a combined indicator of overall morbidity and mortality in the population, maintained a consistently negative association with COVID-19 vaccination levels—over time and across samples, which we interpret as showing vaccination to be a main determinant of DALYs, rather than the other way around. In terms of density of medical personnel, our results rule out number of medical doctors (MDs) relative to population size as an important factor, against the background of previous research that has found a positive relation (44, 45). It has been argued that GPs in particular were instrumental for vaccine acceptance through “counseling and building local community trust” (45). According to our analysis, perhaps this important role was assumed by other medical staff such as nurses and midwives, pharmacists, and even surgeons, all of whom had a positive influence on vaccine uptake that increased with time. Number of nurses and midwives specifically has received a lot of research attention to mostly positive results (15, 33, 73). A remarkable finding was the very strong positive relation of psychiatrists density on Y1 in OECD countries. The more general variable of total health and social employment also showed a strong positive effect, but only for Y3. While routine childhood vaccination has been found to be itself negatively impacted by the pandemic in many places around the world (62), we were interested in whether pre-pandemic inoculation levels were related to COVID-19 vaccination. The answer was “not much” in our full sample as well as the EU [results consistent with some previous research (34, 60)]. We reason the novelty of the COVID-19 disease and vaccines triggered largely different societal mechanisms when compared with routine childhood immunization. However, for the OECD sub-sample and Y1 in particular, we found a strong negative relation of DPT coverage rate and a strong positive one of the MCV1—effects we currently cannot explain. With regards to expenditure indicators, despite previous evidence of the beneficial effect of health expenditure on COVID-19 vaccination (16, 34, 73), neither this covariate (values for 2019 considered here) nor the one reflecting social expenditure could be named among the important determinants in our analysis, lest for a small to moderate negative effect of the latter on Y3 in the full and OECD samples. It may be that in European and OECD countries on the whole, general wealth and health and social funding have reached a level at which additional increases make less of a difference over a relatively short time period. R&D expenditure though clearly showed a positive influence at the start of the vaccination effort, perhaps reflecting forward-thinking and a strive for and embrace of innovation.

Last but not least, we turn to culture. In our analysis, the six Hofstede indices that we considered, collectively, turned out to be the strongest influence on COVID-19 vaccine uptake in the EU sub-sample, although this was certainly not the case for the full-country and the OECD samples. Starting with individualism (v collectivism), we found this to be the strongest positive determinant for Y1 in the EU, with the effect remaining relatively high for Y2, but disappearing for the booster dose (Y3). This seems counter-intuitive, as one would expect collectivistic societies to be the ones to prioritize common goals such as population health, and indeed, for OECD countries, even if relatively weak, we see a negative association between individualism and vaccination rates throughout the time period under investigation. However, a state-wise study in the US (60) and another multi-country study (63) both found the same results as our EU findings, when considering vaccination with at least one dose (our Y1) around the same time we measured it—individualism was a positive influence on vaccination rate, at least at the beginning of the campaign. On this individualism-collectivism divide, the literature provides less surprising evidence (especially from the first few months of the pandemic) when it comes to compliance with and support for protective measures, where higher national level individualism was found to be negatively related (64) and higher collectivism—positively (65, 66). Following logically, in terms of other COVID-19 outcomes such as cases and deaths, more individualistic cultures were found to have higher numbers of both (67, 68). Previous research is less in agreement on the role of indulgence though, which was linked to higher numbers of COVID-19 cases (67), but also better social distancing behavior (69). Indulgent nations put value on following one's impulses with regards to having fun and enjoying freedom, which does not seem very compatible with voluntary vaccination—a generally unpleasant and inconvenient activity with no immediate, relatively uncertain gratification. Therefore, the negative association we found between this cultural index and Y1 in the full sample, but especially in the OECD sub-sample (in fact, the strongest overall effect for Y1 in OECD countries) seems to makes sense. This relationship lessens in magnitude for OECD countries, but it remains negative, while it actually changes sign for Y3 in the full sample, to a relatively small positive value. This might be explained by the older and more vulnerable population—the prime target of booster campaigns captured in our Y3 outcome by June 2022—being more eager to receive booster vaccination to protect themselves in nations where the rest of the population is returning quicker to pre-pandemic social mixing after they have received a primary series of vaccination. Interestingly, a positive association between indulgence levels and vaccination with at least one dose was found in other research (56, 63), where it was hypothesized that “indulgent societies might have a greater desire to restore normalcy… and are therefore more likely to get vaccinated” (56). The national culture aspect of uncertainty avoidance is another relevant factor. Given the overarching uncertainty and potential long-term economic and political instability the pandemic stirred, it could be expected that societies characterized by higher uncertainty avoidance tendencies would do the necessary to end the pandemic sooner—hence, embrace vaccination. Our results certainly tell this story for EU countries, and, to a lesser degree for all 61 relatively wealthy nations we included in the analysis (however, this index was not a significant factor for OECD vaccination). There is some support in the literature to our finding of a positive influence of uncertainty avoidance on vaccine uptake (63), while its relation to other COVID-19 outcomes and NPIs is less clear (64, 70, 71). Furthermore, the long-term orientation covariate provided another set of surprising results, as one would think countries high on this national culture trait are invested in ensuring the future wellbeing of the nation and its people. Although the effect in our results was quite small, in relative terms, we see a definite negative relation between this index and vaccination. This counter-intuitive finding though goes hand in hand with existing evidence that long-term orientation is associated with lower support for NPIs and higher COVID-19 cases and deaths (64, 69, 70). Power distance, or the acceptance of the unequal distribution of power, showed a somewhat consistent negative effect throughout the vaccination effort, especially noticeable in the EU-only part of the analysis. This is another hard-to-explain relationship that warrants further investigation into the subtle cultural influences in times of health crises. Finally, masculinity was the least significant cultural trait in our analysis, with a relatively weak negative association to vaccination in the full sample, a relatively weak positive one in the EU, and nearly no influence in the OECD sub-sample.

## 5 Limitations and conclusions

The main limitation our study faces stems from questionable data quality and reliability for some of the investigated covariates. Unfortunately, not all countries in our selection have up-to-date data on two very important healthcare indicators in the context of the pandemic—ICU and hospital bed counts. We had to rely on what was available and sometimes these were values recorded as far back as 10 years prior. With regards to tracking COVID-19 disease burden and mortality, different countries employed different (and sometimes changing) reporting schedules—daily, weekly, weekdays only, Monday-Wednesday-Friday, seemingly random days, etc.. This necessitated our use of linear interpolation to obtain more precise estimates and in the case of the ICU occupancy covariate it meant that our calculations were based on varying amounts of information depending on country. Lastly, a large number of missing longitudinal data records was problematic for the devising of the “mandatory vaccination” variable. We reckoned it may be a very important one to consider though, so we resorted to making the assumption that missing data signified a value of “0”, or that no requirement for vaccination was in place on that particular day (indeed, for a lot of countries only values of “1” were occasionally recorded and no “0”'s at all).

In order to be able to work with so many variables with moderate levels of missing data, we needed to re-scale all continuous variables, which prevents us from having a direct quantitative interpretation of the size of the effects we have discovered. On the other hand, we are now able to judge strength and importance of each factor in relation to others. We employed the PLSR technique that is often used in order to uncover underlying latent variables. However, as most of our covariates measured very tangible national characteristics, we were not so much concerned with this “classical” part of the output, where resulting latent variables were practically impossible to interpret in a meaningful way. We were, instead, interested in the regression estimates for our original (albeit, standardized) covariates, in order to identify the key country-level factors related to COVID-19 vaccination rates. As PLSR does not produce standard errors for its regression coefficients though, it was technically impossible to formally apply Rubin's rules (72) for pooling the results after MI. We have used 95% confidence intervals around the estimated means over the 100 imputations in order to judge statistical significance (as shown in Appendix B). In practice, all mean estimates of more than 0.05 absolute value can be considered statistically significant based on this criterion.

On a topic with huge implications for the handling of possible future pandemics, with relatively limited research conducted as yet, we investigated a large number of macro-level determinants using statistically sound methods in order to identify the most important drivers behind national COVID-19 vaccination success in relatively wealthy countries. Considering results from all three separate analyses we performed, we can point to the stringency of restrictive measures, higher proportion of older adults, females, and residents living in urban areas as consistently contributing toward higher vaccination rates, while the majority of other factors varied in importance depending on the stage of the vaccination campaign and sample of countries. Overall, results from the full sample of 61 European and OECD countries, and those from the OECD-only sample were more similar, while the EU-only analysis yielded considerably different patterns of influence. While the positive role of healthcare factors (including work force, material base, expenditure, and coverage) was much less prominent in the EU, cultural factors were found to be particularly important for COVID-19 vaccine uptake in this sub-sample. For policy makers, our work provides a thorough basis for understanding between-country differences in evolving uptake of vaccine doses during the COVID-19 pandemic, and can, therefore, serve as a basis to understand the strengths and limitations of specific policies to stimulate uptake during future pandemics. When thinking about pandemic management, high and timely vaccine uptake can be considered as an indicator of success of such management. We showed that slowly evolving demographic and socio-cultural country contexts have important explanatory value for observed vaccine uptake over different pandemic stages. It is in this country context that policy should be formulated during a crisis and evaluated after it.

## Data Availability

Publicly available datasets were analyzed in this study. This data can be found here: Hyperlinks provided in the article.

## Footnotes

^1^ https://github.com/owid/covid-19-data/tree/master/public/data

## References

[1] World Health Organization. WHO Coronavirus (COVID-19) Dashboard. (2023). Available: https://covid19.who.int/ (accessed November 6, 2023).

[2] Msemburi W Karlinsky A Knutson V Aleshin-Guendel S Chatterji S Wakefield J. The WHO estimates of excess mortality associated with the COVID-19 pandemic. Nature. (2023) 613:130–7. 10.1038/s41586-022-05522-2 36517599 PMC9812776

[3] EUROSTAT. Demography 2023 Edition. (2023). Available at: https://ec.europa.eu/eurostat/web/interactive-publications/demography-2023 (accessed November 6, 2023).

[4] Schöley J Aburto JM Kashnitsky I Kniffka MS Zhang L Jaadla H. Life expectancy changes since COVID-19. Nat Hum Behav. (2022) 6:1649–59. 10.1038/s41562-022-01450-3 36253520 PMC9755047

[5] Iftekhar EN Priesemann V Balling R Bauer S Beutels P Calero Valdez A. A look into the future of the COVID-19 pandemic in Europe: an expert consultation. Lancet Reg Health. (2021) 8:100185. 10.1016/j.lanepe.2021.100185 34345876 PMC8321710

[6] Liu Y Sandmann FG Barnard RC Pearson CAB Pastore R Pebody R. Optimising health and economic impacts of COVID-19 vaccine prioritisation strategies in the WHO European Region: a mathematical modelling study. Lancet Reg Health. (2022) 12:100267. 10.1016/j.lanepe.2021.100267 34870256 PMC8629724

[7] Khatatbeh M Albalas S Khatatbeh H Momani W Melhem O Al Omari O. Children's rates of COVID-19 vaccination as reported by parents, vaccine hesitancy, and determinants of COVID-19 vaccine uptake among children: a multi-country study from the Eastern Mediterranean Region. BMC Public Health. (2022) 22:1375. 10.1186/s12889-022-13798-2 35850675 PMC9294741

[8] Serajuddin U Hamadeh N. New World Bank Country Classifications by Income Level: 2020-2021. (2020). Available: https://blogs.worldbank.org/opendata/new-world-bank-country-classifications-income-level-2020-2021 (accessed February 9, 2023).

[9] Jit M Ananthakrishnan A Mckee M Wouters OJ Beutels P Teerawattananon Y. Multi-country collaboration in responding to global infectious disease threats: lessons for Europe from the COVID-19 pandemic. Lancet Reg Health. (2021) 9:100221. 10.1016/j.lanepe.2021.100221 34642675 PMC8495250

[10] Hale T Angrist N Goldszmidt R Kira B Petherick A Phillips T. A global panel database of pandemic policies (Oxford COVID-19 Government Response Tracker). Nat Hum Behav. (2021) 5:529–38. 10.1038/s41562-021-01079-8 33686204

[11] van Buuren SGO-K. mice: multivariate imputation by chained equations in R. J Stat Softw. (2011) 45:i03. 10.18637/jss.v045.i03

[12] Wold H. Estimation of principal components and related models by iterative least squares: Sonderdruck aus. In:R. Krishnaiah Paruchuri, editor. Multivariate Analysis, London; New York, NY: Academic Press ohne Jahr (1966). p. 391–420.

[13] Wold S Sjöström M Eriksson L. PLS-regression: a basic tool of chemometrics. Chemometr Intell Lab Syst. (2001) 58:109–30. 10.1016/S0169-7439(01)00155-138384681

[14] Sawatsky ML Clyde M Meek F. Partial least squares regression in the social sciences. Quant Methods Psychol. (2015) 11:52–62. 10.20982/tqmp.11.2.p052

[15] Tehrani SO Perkins DD. Community health resources, globalization, trust in science, and voting as predictors of COVID-19 vaccination rates: a global study with implications for vaccine adherence. Vaccines. (2022) 10:1343. 10.3390/vaccines10081343 36016231 PMC9416245

[16] Teperowski Monrad J Quaade S Powell-Jackson T. Supply, then demand? Health expenditure, political leanings, cost obstacles to care, and vaccine hesitancy predict state-level COVID-19 vaccination rates. Vaccine. (2022) 40:6528–48. 10.1016/j.vaccine.2022.08.050 36202641 PMC9452439

[17] Vaidya V Partha G Karmakar M. Gender differences in utilization of preventive care services in the United States. J Womens Health. (2012) 21:140–5. 10.1089/jwh.2011.2876 22081983

[18] Brown GD Largey A Mcmullan C. The impact of gender on risk perception: Implications for EU member states' national risk assessment processes. Int J Disast Risk Reduct. (2021) 63:102452. 10.1016/j.ijdrr.2021.102452

[19] Capraro V Barcelo HLN. The effect of messaging and gender on intentions to wear a face covering to slow down COVID-19 transmission. J Behav Econ Policy. (2020) 4:45–55. 10.31234/osf.io/tg7vz

[20] Zettler I Schild C Lilleholt L Kroencke L Utesch T Moshagen M. The role of personality in COVID-19-related perceptions, evaluations, and behaviors: findings across five samples, nine traits, and 17 criteria. Soc Psychol Personal Sci. (2022) 13:299–310. 10.1177/19485506211001680

[21] Galasso V Pons V Profeta P Becher M Brouard S Foucault M. Gender differences in COVID-19 attitudes and behavior: panel evidence from eight countries. Proc Natl Acad Sci USA. (2020) 117:27285–91. 10.1073/pnas.2012520117 33060298 PMC7959517

[22] Boutsikari EC Christakou A Elpidoforou M Kopsidas I Nikolovienis N Kardara D. Greek population's perceptions of nonpharmacological interventions towards the first wave of COVID-19 pandemic mitigation: a regressionbased association analysis. Pneumon. (2021) 34:1–11. 10.18332/pne/141592

[23] Okten IO Gollwitzer A Oettingen G. Gender differences in preventing the spread of coronavirus. Behav Sci Policy. (2020) 6:109–22. 10.1177/237946152000600214

[24] Otterbring T Festila A. Pandemic prevention and personality psychology: gender differences in preventive health behaviors during COVID-19 and the roles of agreeableness and conscientiousness. J Saf Sci Resil. (2022) 3:87–91. 10.1016/j.jnlssr.2021.11.003 PMC8639385 40477760

[25] Pedersen MJ Favero N. Social distancing during theCOVID-19 pandemic: who are the present and future noncompliers? Public Adm Rev. (2020) 80:805–14. 10.1111/puar.13240 32836442 PMC7280647

[26] Sobol M Blachnio A Przepiórka A. Time of pandemic: temporal perspectives related to compliance with public health regulations concerning the COVID-19 pandemic. Soc Sci Med. (2020) 265:113408. 10.1016/j.socscimed.2020.113408 33045654 PMC7537628

[27] Vardavas CI Odani S Nikitara K El Banhawi H Kyriakos CN Taylor L. Perceptions and practice of personal protective behaviors to prevent COVID-19 transmission in the G7 nations. Popul Med. (2020) 2:123821. 10.18332/popmed/123821

[28] Aschwanden D Strickhouser JE Sesker AA Lee JH Luchetti M Stephan Y. Psychological and behavioural responses to Coronavirus disease 2019: the role of personality. Eur J Pers. (2021) 35:51–66. 10.1002/per.2281 32836766 PMC7361622

[29] Carvalho LF Pianowski G Gonçalves AP. Personality differences and COVID-19: are extroversion and conscientiousness personality traits associated with engagement with containment measures? Trends Psychiatry Psychother. (2020) 42:179–84. 10.1590/2237-6089-2020-0029 32294713

[30] Han H. Exploring the association between compliance with measures to prevent the spread of COVID-19 and big five traits with Bayesian generalized linear model. Pers Individ Dif. (2021) 176:110787. 10.1016/j.paid.2021.110787 33642661 PMC7901385

[31] Nofal AM Cacciotti G Lee N. Who complies with COVID-19 transmission mitigation behavioral guidelines? PLoS ONE. (2020) 15:e0240396. 10.1371/journal.pone.0240396 33031476 PMC7544078

[32] Zajenkowski M Jonason PK Leniarska M Kozakiewicz Z. Who complies with the restrictions to reduce the spread of COVID-19?: Personality and perceptions of the COVID-19 situation. Person Ind Differ. (2020) 166:110199. 10.1016/j.paid.2020.110199 32565591 PMC7296320

[33] Farzanegan MR Hofmann HP. Effect of public corruption on the COVID-19 immunization progress. Sci Rep. (2021) 11:23423. 10.1038/s41598-021-02802-1 34873212 PMC8648879

[34] Pattanshetty S Pardesi M Gudi N. A comparative analysis on the social determinants of COVID-19 vaccination coverage in fragile and conflict affected settings (FCS) and non- fragile and conflict affected settings. Int J Health Policy Manag. (2022) 12:6830. 10.34172/ijhpm.2022.6830 36300252 PMC10125044

[35] Munir H Munir SR. Perceiving freedom: civil liberties and COVID-19 vaccinations. Polit Stud Rev. (2023) 21:190–209. 10.1177/14789299221082460 37038605 PMC10076963

[36] Natalia YA Delporte M De Witte D Beutels P Dewatripont M Molenberghs G. Assessing the impact of COVID-19 passes and mandates on disease transmission, vaccination intention, and uptake: a scoping review. BMC Public Health. (2023) 23:2279. 10.1186/s12889-023-17203-4 37978472 PMC10656887

[37] Goel RK Nelson MA Goel VY. COVID-19 vaccine rollout-scale and speed carry different implications for corruption. J Policy Model. (2021) 43:503–20. 10.1016/j.jpolmod.2021.04.003 33967361 PMC8095025

[38] Karabulut G Zimmermann KF Bilgin MH Doker AC. Democracy and COVID-19 outcomes. Econ Lett. (2021) 203:109840. 10.1016/j.econlet.2021.109840 33814654 PMC7997903

[39] Mazzucchelli R Dieguez AA Costa EMD Villarías NC. Democracy and Covid-19 mortality in Europe. Rev Esp Salud Publica. (2020) 94:e202006073. 32576811 PMC11582997

[40] Yao L Li MH Wan JY Howard SC Bailey JE Graff JC. Democracy and case fatality rate of COVID-19 at early stage of pandemic: a multicountry study. Environ Sci Pollut Res. (2022) 29:8694–704. 10.1007/s11356-021-16250-x 34490579 PMC8421237

[41] Franic J. What lies behind substantial differences in COVID-19 vaccination rates between EU member states? Front Public Health. (2022) 10. 10.3389/fpubh.2022.858265 35757613 PMC9231480

[42] Aida T Shoji M. Cross-country evidence on the role of national governance in boosting COVID-19 vaccination. BMC Public Health. (2022) 22:576. 10.1186/s12889-022-12985-5 35321676 PMC8941364

[43] Baghbanzadeh M Smith M Pilz J Rahman MS Karamehic-Muratovic A Garg A. Country-level governance indicators as predictors of COVID-19 morbidity, mortality, and vaccination coverage: an exploratory global analysis. Am J Trop Med Hyg. (2022) 107:1066–73. 10.4269/ajtmh.22-0107 36318889 PMC9709024

[44] Deb P Furceri D Jimenez D Kothari S Ostry JD Tawk N. Determinants of COVID-19 vaccine rollouts and their effects on health outcomes. Appl Health Econ Health Policy. (2023) 21:71–89. 10.1007/s40258-022-00757-6 36100820 PMC9470512

[45] Peano A Politano G Gianino MM. Determinants of COVID-19 vaccination worldwide: WORLDCOV, a retrospective observational study. Front Public Health. (2023) 11:8612. 10.3389/fpubh.2023.1128612 37719735 PMC10501313

[46] Petkov M Cohen D. Diagnosing Corruption in Healthcare. London: Transparency International (2016).

[47] Lio MC Lee MH. Corruption costs lives: a cross-country study using an IV approach. Int J Health Plann Manag. (2016) 31:175–90. 10.1002/hpm.2305 26122874

[48] Li Q An L Xu J Baliamoune-Lutz M. Corruption costs lives: evidence from a cross-country study. Eur J Health Econ. (2018) 19:153–65. 10.1007/s10198-017-0872-z 28197784

[49] Transparency International. The ABCs of the CPI: How the Corruption Perceptions Index is Calculated. (2021). Available at: https://www.transparency.org/en/news/how-cpi-scores-are-calculated (accessed February 4, 2023).

[50] Peerthum S Luckho T. Exploring the linkage between public corruption and political trust in Mauritius: a PLS-SEM approach. Public Org Rev. (2021) 21:317–35. 10.1007/s11115-020-00491-4

[51] Wang C-H. Government performance, corruption, and political trust in East Asia^*^. Soc Sci Q. (2016) 97:211–31. 10.1111/ssqu.12223

[52] Mipatrini D Stefanelli P Severoni S Rezza G. Vaccinations in migrants and refugees: a challenge for European health systems. A systematic review of current scientific evidence. Pathog Glob Health. (2017) 111:59–68. 10.1080/20477724.2017.1281374 28165878 PMC5375618

[53] ECDPA (2021). Reducing COVID-19 Transmission and Strengthening Vaccine Uptake Among Migrant Populations in the EU/EEA. Stockholm: European Centre for Disease Prevention and Control.

[54] Fano V Coviello E Consonni D Agresta A Orsini N Crielesi A. COVID-19 vaccines coverage and effectiveness against SARS-CoV-2 infection among residents in the largest Health Authority of Lazio region (Italy): a population-based cohort study. Expert Rev Vacc. (2022) 21:1147–57. 10.1080/14760584.2022.2080057 35584901

[55] Kraft KB Godøy AA Vinjerui KH Kour P Kjøllesdal MKR Indseth T. COVID-19 vaccination coverage by immigrant background. Tidsskr Nor Laegeforen. (2022) 141. 10.4045/tidsskr.21.0799 35107952

[56] Trepanowski R Drazkowski D. Cross-national comparison of religion as a predictor of COVID-19 vaccination rates. J Relig Health. (2022) 61:2198–211. 10.1007/s10943-022-01569-7 35556198 PMC9095816

[57] Varbanova V Beutels P. Recent quantitative research on determinants of health in high income countries: a scoping review. PLoS ONE. (2020) 15:e0239031. 10.1371/journal.pone.0239031 32941493 PMC7498048

[58] Varbanova V Hens N Beutels P. Determinants of life-expectancy and disability-adjusted life years (DALYs) in European and Organisation for Economic Co-operation and Development (OECD) countries: A longitudinal analysis (1990-2019). SSM Popul Health. (2023) 24:101484. 10.1016/j.ssmph.2023.101484 37680998 PMC10480329

[59] Varbanova V Verelst F Hens N Beutels P. Determinants of basic childhood vaccination coverage in European and OECD countries. Hum Vaccin Immunother. (2022) 18:2123883. 10.1080/21645515.2022.2123883 36173818 PMC9746410

[60] Martens JP Rutjens BT. Spirituality and religiosity contribute to ongoing COVID-19 vaccination rates: comparing 195 regions around the world. Vaccine X. (2022) 12:100241. 10.1016/j.jvacx.2022.100241 36407820 PMC9666266

[61] Verelst F Kuylen E Beutels P. Indications for healthcare surge capacity in European countries facing an exponential increase in coronavirus disease (COVID-19) cases, March 2020. Eurosurveillance. (2020) 25:2000323. 10.2807/1560-7917.ES.2020.25.13.2000323 32265003 PMC7140594

[62] Ghaznavi C Eguchi A Suu Lwin K Yoneoka D Tanoue Y Kumar Rauniyar S. Estimating global changes in routine childhood vaccination coverage during the COVID-19 pandemic, 2020–2021. Vaccine. (2023) 41:4151–7. 10.1016/j.vaccine.2023.05.034 37246068 PMC10201316

[63] Lajunen T Gaygisiz E Gaygisiz Ü. Socio-cultural correlates of the COVID-19 outcomes. J Epidemiol Glob Health. (2022) 12:328–39. 10.1007/s44197-022-00055-3 35997899 PMC9395878

[64] Lucas T Manning M Strelan P Kopetz C Agostini M Bélanger JJ. Justice beliefs and cultural values predict support for COVID-19 vaccination and quarantine behavioral mandates: a multilevel cross-national study. Transl Behav Med. (2022) 12:284–90. 10.1093/tbm/ibab153 35038333 PMC8807214

[65] Cho H Guo Y Torelli C. Collectivism fosters preventive behaviors to contain the spread of COVID-19: implications for social marketing in public health. Psychol Mark. (2022) 39:694–700. 10.1002/mar.21613 35465078 PMC9015240

[66] Lu JG Jin P English AS. Collectivism predicts mask use during COVID-19. Proc Natl Acad Sci USA. (2021) 118:e2021793118. 10.1073/pnas.2021793118 34016707 PMC8201834

[67] Gokmen Y Baskici C Ercil Y. The impact of national culture on the increase of COVID-19: a cross-country analysis of European countries. Int J Intercult Relat. (2021) 81:1–8. 10.1016/j.ijintrel.2020.12.006 33518841 PMC7833793

[68] Windsor LC Yannitell Reinhardt G Windsor AJ Ostergard R Allen S Burns C. Gender in the time of COVID-19: evaluating national leadership and COVID-19 fatalities. PLoS ONE. (2020) 15:e0244531. 10.1371/journal.pone.0244531 33382791 PMC7774849

[69] Wang Y. Government policies, national culture and social distancing during the first wave of the COVID-19 pandemic: international evidence. Saf Sci. (2021) 135:105138. 10.1016/j.ssci.2020.105138 36570788 PMC9759369

[70] Erman A Medeiros M. Exploring the effect of collective cultural attributes on Covid-19-related public health outcomes. Front Psychol. (2021) 12:627669. 10.3389/fpsyg.2021.627669 33833717 PMC8021731

[71] Huynh TLD. Does culture matter social distancing under the COVID-19 pandemic? Saf Sci. (2020) 130:104872. 10.1016/j.ssci.2020.104872 32550745 PMC7284251

[72] Rubin DB. Multiple Imputation for Nonresponse in Surveys. New York, NY: Wiley (1987).

[73] Omidvar Tehrani S Perkins DD. Public health resources, religion, and freedom as predictors of COVID-19 vaccination rates: a global study of 89 countries. COVID. (2022) 2:703–18. 10.3390/covid2060053 PMC9416245 36016231

