# Supplementary material for: Determinants of COVID-19 vaccination coverage in European and Organisation for Economic Co-operation and Development (OECD) countries
Source: Front Public Health. 2024 Dec 31;12:1466858. doi: 10.3389/fpubh.2024.1466858 (PMC11735944; doi:10.3389/fpubh.2024.1466858)
Supplement: Supplementary file 1 [file Data_Sheet_1.docx]

Table. Ranking of countries included in the analysis according to gross national income (GNI) per capita (in current USD) for 2019 (data not available for Andorra, Liechtenstein, Monaco, San Marino). The World Bank’s classification by income level from the 1^st^ of July 2020 shown is based on GNI per capita for 2019*.

| **Ranking** | **Country** | **GNI** | **OECD** | **Europe** | **EU** |
| --- | --- | --- | --- | --- | --- |
|  | ***high income (>$12,535****)* |  |  |  |  |
| 1 | Switzerland | $84,260 | • | • |  |
| 2 | Norway | $81,640 | • | • |  |
| 3 | Luxembourg | $77,040 | • | • | • |
| 4 | Iceland | $72,900 | • | • |  |
| 5 | USA | $65,970 | • |  |  |
| 6 | Denmark | $63,460 | • | • | • |
| 7 | Ireland | $63,230 | • | • | • |
| 8 | Sweden | $56,410 | • | • | • |
| 9 | Australia | $54,910 | • |  |  |
| 10 | Netherlands | $53,180 | • | • | • |
| 11 | Austria | $50,960 | • | • | • |
| 12 | Finland | $49,940 | • | • | • |
| 13 | Germany | $49,140 | • | • | • |
| 14 | Belgium | $48,010 | • | • | • |
| 15 | Canada | $46,550 | • |  |  |
| 16 | Israel | $43,540 | • |  |  |
| 17 | UK | $43,460 | • |  |  |
| 18 | New Zealand | $42,870 | • |  |  |
| 19 | France | $42,510 | • | • | • |
| 20 | Japan | $42,330 | • |  |  |
| 21 | Italy | $34,870 | • | • | • |
| 22 | South Korea | $33,860 | • |  |  |
| 23 | Spain | $30,350 | • | • | • |
| 24 | Cyprus | $28,600 |  | • | • |
| 25 | Malta | $28,340 |  | • | • |
| 26 | Slovenia | $25,950 | • | • | • |
| 27 | Estonia | $23,250 | • | • | • |
| 28 | Portugal | $23,170 | • | • | • |
| 29 | Czech Republic | $22,110 | • | • | • |
| 30 | Greece | $19,650 | • | • | • |
| 31 | Slovakia | $19,230 | • | • | • |
| 32 | Lithuania | $19,050 | • | • | • |
| 33 | Latvia | $17,790 | • | • | • |
| 34 | Hungary | $16,520 | • | • | • |
| 35 | Poland | $15,360 | • | • | • |
| 36 | Croatia | $15,320 |  | • | • |
| 37 | Chile | $14,990 | • |  |  |
| 38 | Romania | $12,620 |  | • | • |
|  | ***upper-middle income ($4,046 - $12,535****)* |  |  |  |  |
| 39 | Costa Rica | $12,090 | • |  |  |
| 40 | Russia | $11,250 |  | • |  |
| 41 | Turkey | $9,690 | • | • |  |
| 42 | Bulgaria | $9,500 |  | • | • |
| 43 | Mexico | $9,470 | • |  |  |
| 44 | Montenegro | $9,130 |  | • |  |
| 45 | Kazakhstan | $8,820 |  | • |  |
| 46 | Serbia | $7,040 |  | • |  |
| 47 | Colombia | $6,570 | • |  |  |
| 48 | Belarus | $6,370 |  | • |  |
| 49 | Bosnia and Herzegovina | $6,180 |  | • |  |
| 50 | North Macedonia | $5,890 |  | • |  |
| 51 | Albania | $5,230 |  | • |  |
| 52 | Georgia | $4,690 |  | • |  |
| 53 | Armenia | $4,660 |  | • |  |
| 54 | Kosovo | $4,640 |  | • |  |
| 55 | Moldova | $4,580 |  | • |  |
| 56 | Azerbaijan | $4,510 |  | • |  |
|  | ***lower-middle income ($1,036 - $4,045****)* |  |  |  |  |
| 57 | Ukraine | $3,310 |  | • |  |

*** [*https://blogs.worldbank.org/opendata/new-world-bank-country-classifications-income-level-2020-2021*](https://blogs.worldbank.org/opendata/new-world-bank-country-classifications-income-level-2020-2021)

*EU – country is a member of the European Union*

*OECD – country is a member of the Organisation for Economic Co-operation and Development*
