# Supplementary material for: Determinants of COVID-19 vaccination coverage in European and Organisation for Economic Co-operation and Development (OECD) countries
Source: Front Public Health. 2024 Dec 31;12:1466858. doi: 10.3389/fpubh.2024.1466858 (PMC11735944; doi:10.3389/fpubh.2024.1466858)
Supplement: Supplementary file 2 [file Data_Sheet_2.docx]

*Table B1. Partial least squares regression (PLSR) pooled estimates for Y1 in the full sample of 61 European and OECD countries (covariates in bold considered to have statistically significant effects due to 95% confidence interval not containing 0; covariates listed in alphabetical order)*

| **covariate** | **mean** | **95% confidence interval lower boundary** | **95% confidence interval upper boundary** | **standard error** | **standard deviation** | **minimum value** | **maximum value** |
| --- | --- | --- | --- | --- | --- | --- | --- |
| **alcohol consumption** | -0.044 | -0.076 | -0.011 | 0.016 | 0.163 | -0.581 | 0.391 |
| **area** | 0.045 | 0.024 | 0.066 | 0.010 | 0.105 | -0.187 | 0.257 |
| **birth rate** | -0.053 | -0.084 | -0.021 | 0.016 | 0.160 | -0.355 | 0.463 |
| **corruption perception** | 0.240 | 0.198 | 0.282 | 0.021 | 0.211 | -0.325 | 0.719 |
| **DALYs** | -0.188 | -0.227 | -0.149 | 0.020 | 0.196 | -0.683 | 0.352 |
| **democracy** | -0.166 | -0.200 | -0.132 | 0.017 | 0.171 | -0.526 | 0.306 |
| **difference in GRI1** | -0.034 | -0.056 | -0.011 | 0.011 | 0.115 | -0.272 | 0.250 |
| **DPT immunization** | -0.032 | -0.058 | -0.007 | 0.013 | 0.127 | -0.366 | 0.375 |
| **economic freedom** | -0.200 | -0.231 | -0.169 | 0.016 | 0.158 | -0.706 | 0.182 |
| education | -0.007 | -0.039 | 0.024 | 0.016 | 0.159 | -0.368 | 0.635 |
| excess mortality1 | 0.004 | -0.029 | 0.038 | 0.017 | 0.169 | -0.367 | 0.498 |
| **Facebook audience** | 0.169 | 0.132 | 0.205 | 0.018 | 0.183 | -0.312 | 0.530 |
| **female labor** | -0.102 | -0.132 | -0.072 | 0.015 | 0.152 | -0.592 | 0.293 |
| **female pop** | 0.235 | 0.193 | 0.277 | 0.021 | 0.211 | -0.331 | 0.778 |
| **GDP** | 0.054 | 0.021 | 0.087 | 0.016 | 0.164 | -0.377 | 0.503 |
| **gini** | -0.081 | -0.109 | -0.053 | 0.014 | 0.142 | -0.398 | 0.419 |
| **GRInon-vaccinated1** | 0.403 | 0.383 | 0.424 | 0.010 | 0.103 | 0.144 | 0.696 |
| **health and social employment** | 0.051 | 0.010 | 0.091 | 0.020 | 0.203 | -0.335 | 0.590 |
| **health expenditure** | -0.105 | -0.138 | -0.072 | 0.017 | 0.167 | -0.678 | 0.206 |
| **hospital density** | -0.097 | -0.120 | -0.073 | 0.012 | 0.120 | -0.437 | 0.189 |
| **hospital beds** | -0.386 | -0.424 | -0.348 | 0.019 | 0.192 | -1.064 | 0.044 |
| hospital employment | -0.010 | -0.049 | 0.028 | 0.019 | 0.192 | -0.457 | 0.466 |
| **ICU occupancy1** | -0.048 | -0.074 | -0.022 | 0.013 | 0.132 | -0.315 | 0.270 |
| **individualism** | 0.049 | 0.015 | 0.083 | 0.017 | 0.172 | -0.356 | 0.535 |
| **indulgence** | -0.108 | -0.140 | -0.077 | 0.016 | 0.159 | -0.549 | 0.276 |
| **long-term care residents** | 0.048 | 0.024 | 0.072 | 0.012 | 0.122 | -0.186 | 0.343 |
| long-term orientation | -0.008 | -0.037 | 0.021 | 0.015 | 0.147 | -0.471 | 0.314 |
| **mandatory vaccination1** | -0.449 | -0.485 | -0.413 | 0.018 | 0.183 | -0.918 | -0.010 |
| **masculinity** | -0.030 | -0.051 | -0.008 | 0.011 | 0.109 | -0.335 | 0.328 |
| MCV1 immunization | -0.029 | -0.058 | 0.000 | 0.014 | 0.145 | -0.465 | 0.298 |
| **MDs density** | -0.118 | -0.142 | -0.093 | 0.012 | 0.122 | -0.429 | 0.197 |
| **nurses and midwives density** | -0.029 | -0.056 | -0.002 | 0.014 | 0.137 | -0.364 | 0.346 |
| **power distance** | -0.082 | -0.115 | -0.048 | 0.017 | 0.170 | -0.544 | 0.397 |
| **pharmacists density** | 0.022 | 0.002 | 0.042 | 0.010 | 0.100 | -0.256 | 0.217 |
| **population density** | 0.053 | 0.020 | 0.086 | 0.017 | 0.166 | -0.462 | 0.513 |
| **population growth** | 0.133 | 0.101 | 0.165 | 0.016 | 0.161 | -0.522 | 0.539 |
| population size | -0.023 | -0.056 | 0.010 | 0.017 | 0.166 | -0.421 | 0.378 |
| **population 15-64yrs** | 0.106 | 0.081 | 0.131 | 0.013 | 0.126 | -0.244 | 0.496 |
| **population 65+** | 0.481 | 0.448 | 0.513 | 0.016 | 0.162 | 0.089 | 0.901 |
| poverty | -0.020 | -0.052 | 0.012 | 0.016 | 0.162 | -0.505 | 0.450 |
| **psychiatrists density** | 0.087 | 0.060 | 0.115 | 0.014 | 0.138 | -0.315 | 0.456 |
| **political stability and lack of violence/terrorism** | 0.183 | 0.148 | 0.218 | 0.018 | 0.178 | -0.209 | 0.659 |
| **R&D expenditure** | 0.226 | 0.192 | 0.260 | 0.017 | 0.171 | -0.177 | 0.674 |
| **right-wing in government** | -0.032 | -0.051 | -0.013 | 0.010 | 0.097 | -0.247 | 0.225 |
| smoking prevalence | 0.024 | -0.005 | 0.052 | 0.014 | 0.143 | -0.420 | 0.462 |
| social expenditure | 0.002 | -0.028 | 0.032 | 0.015 | 0.150 | -0.350 | 0.421 |
| **surgeons density** | 0.067 | 0.038 | 0.096 | 0.015 | 0.146 | -0.323 | 0.391 |
| **total deaths1** | 0.121 | 0.093 | 0.150 | 0.014 | 0.143 | -0.357 | 0.414 |
| **total healthcare coverage** | -0.161 | -0.192 | -0.130 | 0.015 | 0.155 | -0.506 | 0.157 |
| **Twitter audience** | 0.042 | 0.009 | 0.075 | 0.017 | 0.168 | -0.412 | 0.414 |
| **uncertainty avoidance** | 0.099 | 0.070 | 0.128 | 0.015 | 0.148 | -0.257 | 0.411 |
| **unemployment rate** | -0.168 | -0.194 | -0.142 | 0.013 | 0.132 | -0.454 | 0.129 |
| **urban population** | 0.114 | 0.084 | 0.143 | 0.015 | 0.150 | -0.245 | 0.536 |

*Table B2. Partial least squares regression (PLSR) pooled estimates for Y2 in the full sample of 61 European and OECD countries (covariates in bold considered to have statistically significant effects due to 95% confidence interval not containing 0; covariates listed in alphabetical order)*

| **covariate** | **mean** | **95% confidence interval lower boundary** | **95% confidence interval upper boundary** | **standard error** | **standard deviation** | **minimum value** | **maximum value** |
| --- | --- | --- | --- | --- | --- | --- | --- |
| **alcohol consumption** | 0.053 | 0.025 | 0.081 | 0.014 | 0.140 | -0.242 | 0.477 |
| **area** | -0.068 | -0.086 | -0.049 | 0.009 | 0.094 | -0.393 | 0.127 |
| **birth rate** | -0.289 | -0.318 | -0.260 | 0.015 | 0.146 | -0.936 | -0.011 |
| **corruption perception** | 0.116 | 0.077 | 0.155 | 0.020 | 0.196 | -0.382 | 0.578 |
| **DALYs** | -0.152 | -0.188 | -0.115 | 0.018 | 0.182 | -0.681 | 0.246 |
| **democracy** | -0.049 | -0.077 | -0.021 | 0.014 | 0.141 | -0.435 | 0.281 |
| **difference in GRI2** | 0.034 | 0.009 | 0.058 | 0.012 | 0.123 | -0.449 | 0.297 |
| **DPT immunization** | 0.085 | 0.061 | 0.110 | 0.012 | 0.123 | -0.267 | 0.366 |
| **economic freedom** | 0.112 | 0.080 | 0.144 | 0.016 | 0.160 | -0.210 | 0.571 |
| education | -0.012 | -0.040 | 0.016 | 0.014 | 0.142 | -0.562 | 0.356 |
| **excess mortality2** | -0.197 | -0.236 | -0.158 | 0.020 | 0.195 | -0.614 | 0.316 |
| **Facebook audience** | 0.063 | 0.029 | 0.098 | 0.018 | 0.175 | -0.604 | 0.620 |
| **female labor** | -0.116 | -0.149 | -0.083 | 0.017 | 0.166 | -0.556 | 0.414 |
| **female pop** | 0.320 | 0.284 | 0.356 | 0.018 | 0.181 | -0.106 | 0.806 |
| GDP | -0.037 | -0.085 | 0.010 | 0.024 | 0.239 | -0.792 | 0.444 |
| **gini** | 0.051 | 0.021 | 0.080 | 0.015 | 0.148 | -0.481 | 0.386 |
| **GRInon-vaccinated2** | 0.185 | 0.159 | 0.211 | 0.013 | 0.131 | -0.166 | 0.603 |
| **health and social employment** | -0.056 | -0.090 | -0.022 | 0.017 | 0.173 | -0.591 | 0.520 |
| **health expenditure** | -0.071 | -0.103 | -0.040 | 0.016 | 0.159 | -0.544 | 0.291 |
| **hospital density** | 0.028 | 0.000 | 0.056 | 0.014 | 0.140 | -0.344 | 0.483 |
| **hospital beds** | -0.081 | -0.114 | -0.047 | 0.017 | 0.167 | -0.618 | 0.457 |
| **hospital employment** | -0.050 | -0.076 | -0.023 | 0.013 | 0.133 | -0.371 | 0.325 |
| ICU occupancy2 | 0.012 | -0.013 | 0.036 | 0.012 | 0.123 | -0.399 | 0.367 |
| individualism | -0.003 | -0.032 | 0.026 | 0.015 | 0.147 | -0.543 | 0.306 |
| **indulgence** | 0.049 | 0.013 | 0.085 | 0.018 | 0.181 | -0.459 | 0.490 |
| **long-term care residents** | 0.028 | 0.005 | 0.052 | 0.012 | 0.119 | -0.304 | 0.414 |
| **long-term orientation** | -0.064 | -0.096 | -0.033 | 0.016 | 0.160 | -0.519 | 0.262 |
| **mandatory vaccination2** | 0.092 | 0.062 | 0.123 | 0.015 | 0.154 | -0.311 | 0.469 |
| **masculinity** | -0.089 | -0.107 | -0.070 | 0.009 | 0.094 | -0.330 | 0.239 |
| **MCV1 immunization** | -0.083 | -0.105 | -0.061 | 0.011 | 0.112 | -0.401 | 0.184 |
| **MDs density** | -0.054 | -0.082 | -0.026 | 0.014 | 0.140 | -0.425 | 0.230 |
| **nurses and midwives density** | 0.076 | 0.045 | 0.106 | 0.016 | 0.155 | -0.249 | 0.615 |
| **power distance** | -0.094 | -0.119 | -0.069 | 0.013 | 0.126 | -0.472 | 0.176 |
| **pharmacists density** | 0.075 | 0.045 | 0.104 | 0.015 | 0.149 | -0.434 | 0.416 |
| **population density** | -0.137 | -0.179 | -0.095 | 0.021 | 0.214 | -0.641 | 0.368 |
| **population growth** | 0.167 | 0.130 | 0.204 | 0.019 | 0.186 | -0.255 | 0.866 |
| **population size** | 0.106 | 0.072 | 0.140 | 0.017 | 0.170 | -0.318 | 0.587 |
| population 15-64yrs | 0.021 | -0.011 | 0.054 | 0.016 | 0.163 | -0.419 | 0.339 |
| **population 65+** | 0.121 | 0.089 | 0.153 | 0.016 | 0.161 | -0.223 | 0.470 |
| **poverty** | -0.064 | -0.089 | -0.040 | 0.012 | 0.123 | -0.444 | 0.313 |
| **psychiatrists density** | -0.042 | -0.069 | -0.015 | 0.014 | 0.137 | -0.305 | 0.437 |
| **political stability and lack of violence/terrorism** | 0.051 | 0.015 | 0.086 | 0.018 | 0.179 | -0.487 | 0.509 |
| **R&D expenditure** | 0.073 | 0.048 | 0.098 | 0.013 | 0.128 | -0.246 | 0.714 |
| **right-wing in government** | -0.031 | -0.049 | -0.014 | 0.009 | 0.088 | -0.258 | 0.239 |
| smoking prevalence | -0.025 | -0.051 | 0.000 | 0.013 | 0.129 | -0.313 | 0.355 |
| social expenditure | -0.020 | -0.052 | 0.012 | 0.016 | 0.159 | -0.371 | 0.368 |
| **surgeons density** | 0.069 | 0.040 | 0.098 | 0.015 | 0.146 | -0.309 | 0.484 |
| **total deaths2** | -0.136 | -0.166 | -0.105 | 0.015 | 0.154 | -0.547 | 0.181 |
| **total healthcare coverage** | 0.032 | 0.006 | 0.057 | 0.013 | 0.130 | -0.334 | 0.379 |
| **Twitter audience** | 0.049 | 0.017 | 0.081 | 0.016 | 0.162 | -0.390 | 0.462 |
| **uncertainty avoidance** | 0.122 | 0.092 | 0.152 | 0.015 | 0.152 | -0.190 | 0.513 |
| **unemployment rate** | -0.128 | -0.157 | -0.099 | 0.015 | 0.148 | -0.616 | 0.243 |
| **urban population** | 0.106 | 0.079 | 0.134 | 0.014 | 0.138 | -0.309 | 0.384 |

*Table B3. Partial least squares regression (PLSR) pooled estimates for Y3 in the full sample of 61 European and OECD countries (covariates in bold considered to have statistically significant effects due to 95% confidence interval not containing 0; covariates listed in alphabetical order)*

| **covariate** | **mean** | **95% confidence interval lower boundary** | **95% confidence interval upper boundary** | **standard error** | **standard deviation** | **minimum value** | **maximum value** |
| --- | --- | --- | --- | --- | --- | --- | --- |
| **alcohol consumption** | -0.211 | -0.263 | -0.159 | 0.026 | 0.262 | -0.939 | 0.492 |
| **area** | -0.160 | -0.198 | -0.122 | 0.019 | 0.192 | -0.762 | 0.397 |
| **birth rate** | -0.098 | -0.150 | -0.047 | 0.026 | 0.259 | -0.685 | 0.438 |
| **corruption perception** | 0.415 | 0.345 | 0.485 | 0.035 | 0.353 | -0.688 | 1.284 |
| **DALYs** | -0.157 | -0.222 | -0.091 | 0.033 | 0.328 | -0.987 | 0.600 |
| democracy | 0.036 | -0.020 | 0.092 | 0.028 | 0.283 | -0.661 | 0.821 |
| difference in GRI3 | 0.000 | -0.039 | 0.039 | 0.019 | 0.195 | -0.655 | 0.534 |
| **DPT immunization** | -0.063 | -0.118 | -0.007 | 0.028 | 0.277 | -1.029 | 0.600 |
| economic freedom | 0.058 | -0.014 | 0.130 | 0.036 | 0.362 | -0.926 | 0.892 |
| **education** | -0.093 | -0.140 | -0.047 | 0.023 | 0.233 | -0.897 | 0.564 |
| **excess mortality3** | -0.258 | -0.336 | -0.181 | 0.039 | 0.393 | -1.276 | 0.881 |
| **Facebook audience** | -0.098 | -0.165 | -0.031 | 0.034 | 0.337 | -1.193 | 0.656 |
| **female labor** | -0.179 | -0.235 | -0.123 | 0.028 | 0.281 | -0.841 | 0.608 |
| **female pop** | 0.320 | 0.263 | 0.377 | 0.029 | 0.285 | -0.213 | 1.101 |
| **GDP** | -0.158 | -0.223 | -0.093 | 0.033 | 0.328 | -1.127 | 0.631 |
| **gini** | 0.203 | 0.161 | 0.245 | 0.021 | 0.211 | -0.403 | 0.604 |
| **GRInon-vaccinated3** | 0.300 | 0.260 | 0.341 | 0.020 | 0.204 | -0.094 | 0.887 |
| **health and social employment** | 0.220 | 0.161 | 0.278 | 0.029 | 0.294 | -0.596 | 0.970 |
| health expenditure | 0.017 | -0.039 | 0.073 | 0.028 | 0.283 | -0.602 | 0.688 |
| **hospital density** | -0.143 | -0.185 | -0.100 | 0.021 | 0.213 | -0.674 | 0.609 |
| **hospital beds** | 0.127 | 0.066 | 0.188 | 0.031 | 0.308 | -0.456 | 1.106 |
| **hospital employment** | -0.109 | -0.155 | -0.062 | 0.023 | 0.234 | -0.674 | 0.602 |
| **ICU occupancy3** | -0.047 | -0.089 | -0.005 | 0.021 | 0.211 | -0.754 | 0.480 |
| individualism | -0.011 | -0.056 | 0.033 | 0.023 | 0.225 | -0.703 | 0.448 |
| **indulgence** | 0.106 | 0.042 | 0.169 | 0.032 | 0.321 | -0.699 | 1.006 |
| **long-term care residents** | -0.058 | -0.098 | -0.019 | 0.020 | 0.199 | -0.583 | 0.444 |
| **long-term orientation** | -0.166 | -0.216 | -0.116 | 0.025 | 0.252 | -0.783 | 0.430 |
| **mandatory vaccination3** | 0.053 | 0.025 | 0.081 | 0.014 | 0.141 | -0.312 | 0.480 |
| **masculinity** | -0.155 | -0.185 | -0.125 | 0.015 | 0.152 | -0.578 | 0.162 |
| MCV1 immunization | 0.011 | -0.035 | 0.057 | 0.023 | 0.233 | -0.723 | 0.592 |
| MDs density | 0.037 | -0.010 | 0.085 | 0.024 | 0.240 | -0.519 | 0.610 |
| **nurses and midwives density** | 0.119 | 0.075 | 0.163 | 0.022 | 0.222 | -0.536 | 0.764 |
| **power distance** | 0.074 | 0.027 | 0.122 | 0.024 | 0.238 | -0.438 | 0.800 |
| **pharmacists density** | 0.133 | 0.093 | 0.174 | 0.020 | 0.203 | -0.242 | 0.815 |
| **population density** | -0.071 | -0.131 | -0.012 | 0.030 | 0.301 | -0.636 | 0.945 |
| population growth | -0.054 | -0.110 | 0.002 | 0.028 | 0.282 | -0.644 | 0.666 |
| **population size** | -0.218 | -0.273 | -0.164 | 0.028 | 0.276 | -0.895 | 0.286 |
| **population 15-64yrs** | 0.118 | 0.077 | 0.158 | 0.020 | 0.203 | -0.475 | 0.749 |
| **population 65+** | 0.140 | 0.094 | 0.185 | 0.023 | 0.231 | -0.313 | 0.705 |
| **poverty** | -0.122 | -0.159 | -0.086 | 0.018 | 0.185 | -0.565 | 0.412 |
| psychiatrists density | -0.009 | -0.049 | 0.032 | 0.021 | 0.206 | -0.757 | 0.454 |
| **political stability and lack of violence/terrorism** | -0.182 | -0.256 | -0.108 | 0.037 | 0.374 | -1.277 | 0.822 |
| R&D expenditure | -0.023 | -0.066 | 0.020 | 0.022 | 0.217 | -0.569 | 0.679 |
| **right-wing in government** | -0.063 | -0.096 | -0.029 | 0.017 | 0.168 | -0.540 | 0.301 |
| smoking prevalence | -0.019 | -0.071 | 0.034 | 0.026 | 0.263 | -0.781 | 0.570 |
| **social expenditure** | -0.152 | -0.192 | -0.112 | 0.020 | 0.203 | -0.602 | 0.256 |
| **surgeons density** | 0.129 | 0.092 | 0.167 | 0.019 | 0.190 | -0.495 | 0.562 |
| **total deaths3** | 0.158 | 0.110 | 0.206 | 0.024 | 0.242 | -0.496 | 0.819 |
| **total healthcare coverage** | -0.163 | -0.217 | -0.109 | 0.027 | 0.271 | -0.874 | 0.310 |
| **Twitter audience** | 0.111 | 0.059 | 0.164 | 0.026 | 0.264 | -0.485 | 0.798 |
| **uncertainty avoidance** | 0.095 | 0.054 | 0.136 | 0.020 | 0.205 | -0.386 | 0.840 |
| **unemployment rate** | -0.147 | -0.198 | -0.096 | 0.026 | 0.257 | -0.751 | 0.427 |
| **urban population** | 0.137 | 0.087 | 0.187 | 0.025 | 0.254 | -0.583 | 0.886 |

*Table B4. Partial least squares regression (PLSR) pooled estimates for Y1 in EU countries (covariates in bold considered to have statistically significant effects due to 95% confidence interval not containing 0; covariates listed in alphabetical order)*

| **covariate** | **mean** | **95% confidence interval lower boundary** | **95% confidence interval upper boundary** | **standard error** | **standard deviation** | **minimum value** | **maximum value** |
| --- | --- | --- | --- | --- | --- | --- | --- |
| **alcohol consumption** | 0.027 | 0.018 | 0.035 | 0.004 | 0.044 | -0.067 | 0.190 |
| **area** | -0.051 | -0.054 | -0.047 | 0.002 | 0.018 | -0.108 | -0.018 |
| **birth rate** | -0.137 | -0.144 | -0.131 | 0.003 | 0.032 | -0.227 | -0.040 |
| **corruption perception** | 0.070 | 0.063 | 0.077 | 0.003 | 0.035 | -0.026 | 0.144 |
| **DALYs** | -0.112 | -0.122 | -0.102 | 0.005 | 0.049 | -0.343 | -0.008 |
| **democracy** | 0.076 | 0.067 | 0.086 | 0.005 | 0.048 | -0.046 | 0.217 |
| **difference in GRI1** | -0.042 | -0.048 | -0.036 | 0.003 | 0.030 | -0.137 | 0.017 |
| **DPT immunization** | -0.018 | -0.025 | -0.011 | 0.004 | 0.036 | -0.096 | 0.074 |
| **economic freedom** | 0.097 | 0.090 | 0.104 | 0.004 | 0.036 | -0.018 | 0.172 |
| **education** | -0.045 | -0.055 | -0.034 | 0.005 | 0.052 | -0.208 | 0.095 |
| **excess mortality1** | -0.094 | -0.103 | -0.084 | 0.005 | 0.048 | -0.218 | 0.024 |
| **Facebook audience** | 0.126 | 0.115 | 0.136 | 0.005 | 0.053 | 0.018 | 0.312 |
| **female labor** | 0.010 | 0.002 | 0.018 | 0.004 | 0.040 | -0.086 | 0.124 |
| **female pop** | 0.102 | 0.095 | 0.108 | 0.003 | 0.034 | 0.007 | 0.173 |
| GDP | 0.000 | -0.008 | 0.008 | 0.004 | 0.040 | -0.105 | 0.071 |
| **gini** | -0.189 | -0.199 | -0.178 | 0.005 | 0.053 | -0.359 | -0.019 |
| **GRInon-vaccinated1** | 0.075 | 0.068 | 0.081 | 0.003 | 0.032 | -0.003 | 0.158 |
| health and social employment | -0.014 | -0.028 | 0.000 | 0.007 | 0.070 | -0.175 | 0.190 |
| **health expenditure** | 0.020 | 0.013 | 0.027 | 0.004 | 0.035 | -0.111 | 0.090 |
| **hospital density** | -0.018 | -0.030 | -0.007 | 0.006 | 0.057 | -0.173 | 0.123 |
| **hospital beds** | -0.011 | -0.019 | -0.004 | 0.004 | 0.038 | -0.107 | 0.087 |
| **hospital employment** | -0.024 | -0.036 | -0.012 | 0.006 | 0.061 | -0.155 | 0.134 |
| **ICU occupancy1** | 0.049 | 0.040 | 0.057 | 0.004 | 0.042 | -0.094 | 0.140 |
| **individualism** | 0.192 | 0.182 | 0.202 | 0.005 | 0.050 | 0.070 | 0.292 |
| **indulgence** | 0.014 | 0.006 | 0.022 | 0.004 | 0.040 | -0.066 | 0.130 |
| **long-term care residents** | 0.043 | 0.037 | 0.050 | 0.003 | 0.034 | -0.038 | 0.150 |
| **long-term orientation** | 0.010 | 0.000 | 0.019 | 0.005 | 0.046 | -0.152 | 0.111 |
| **mandatory vaccination1** | 0.012 | 0.008 | 0.016 | 0.002 | 0.020 | -0.050 | 0.059 |
| **masculinity** | 0.148 | 0.141 | 0.155 | 0.003 | 0.034 | 0.055 | 0.246 |
| MCV1 immunization | 0.002 | -0.006 | 0.011 | 0.004 | 0.043 | -0.119 | 0.104 |
| **MDs density** | -0.012 | -0.020 | -0.004 | 0.004 | 0.040 | -0.115 | 0.081 |
| nurses and midwives density | 0.000 | -0.010 | 0.010 | 0.005 | 0.050 | -0.132 | 0.156 |
| **power distance** | -0.102 | -0.113 | -0.092 | 0.005 | 0.051 | -0.287 | 0.035 |
| pharmacists density | -0.008 | -0.018 | 0.003 | 0.005 | 0.053 | -0.165 | 0.138 |
| **population density** | -0.006 | -0.009 | -0.003 | 0.002 | 0.016 | -0.050 | 0.037 |
| **population growth** | 0.019 | 0.011 | 0.028 | 0.004 | 0.044 | -0.104 | 0.117 |
| population size | -0.002 | -0.010 | 0.006 | 0.004 | 0.040 | -0.095 | 0.107 |
| **population 15-64yrs** | 0.040 | 0.030 | 0.050 | 0.005 | 0.051 | -0.055 | 0.209 |
| **population 65+** | 0.081 | 0.076 | 0.087 | 0.003 | 0.029 | 0.016 | 0.146 |
| **poverty** | -0.061 | -0.074 | -0.048 | 0.007 | 0.066 | -0.200 | 0.100 |
| psychiatrists density | -0.008 | -0.020 | 0.004 | 0.006 | 0.059 | -0.144 | 0.205 |
| **political stability and lack of violence/terrorism** | 0.055 | 0.050 | 0.060 | 0.003 | 0.025 | -0.010 | 0.143 |
| **R&D expenditure** | 0.033 | 0.027 | 0.039 | 0.003 | 0.031 | -0.059 | 0.110 |
| **right-wing in government** | -0.118 | -0.125 | -0.110 | 0.004 | 0.037 | -0.199 | 0.008 |
| **smoking prevalence** | -0.037 | -0.048 | -0.026 | 0.006 | 0.055 | -0.177 | 0.067 |
| **social expenditure** | 0.078 | 0.067 | 0.089 | 0.006 | 0.056 | -0.057 | 0.266 |
| **surgeons density** | 0.026 | 0.017 | 0.035 | 0.005 | 0.047 | -0.118 | 0.150 |
| **total deaths1** | 0.012 | 0.004 | 0.020 | 0.004 | 0.040 | -0.108 | 0.105 |
| total healthcare coverage | 0.005 | -0.005 | 0.015 | 0.005 | 0.049 | -0.112 | 0.134 |
| **Twitter audience** | 0.032 | 0.024 | 0.040 | 0.004 | 0.039 | -0.107 | 0.098 |
| **uncertainty avoidance** | 0.169 | 0.159 | 0.179 | 0.005 | 0.049 | 0.005 | 0.258 |
| **unemployment rate** | -0.022 | -0.028 | -0.015 | 0.003 | 0.034 | -0.118 | 0.065 |
| **urban population** | 0.130 | 0.124 | 0.136 | 0.003 | 0.031 | 0.052 | 0.208 |

*Table B5. Partial least squares regression (PLSR) pooled estimates for Y2 in EU countries (covariates in bold considered to have statistically significant effects due to 95% confidence interval not containing 0; covariates listed in alphabetical order)*

| **covariate** | **mean** | **95% confidence interval lower boundary** | **95% confidence interval upper boundary** | **standard error** | **standard deviation** | **minimum value** | **maximum value** |
| --- | --- | --- | --- | --- | --- | --- | --- |
| **alcohol consumption** | 0.091 | 0.074 | 0.108 | 0.009 | 0.085 | -0.147 | 0.302 |
| area | -0.008 | -0.016 | 0.000 | 0.004 | 0.039 | -0.124 | 0.121 |
| **birth rate** | -0.056 | -0.068 | -0.043 | 0.006 | 0.062 | -0.215 | 0.118 |
| **corruption perception** | 0.023 | 0.009 | 0.037 | 0.007 | 0.070 | -0.199 | 0.221 |
| **DALYs** | -0.090 | -0.107 | -0.072 | 0.009 | 0.089 | -0.264 | 0.201 |
| **democracy** | -0.093 | -0.109 | -0.076 | 0.008 | 0.083 | -0.422 | 0.079 |
| **difference in GRI2** | -0.020 | -0.032 | -0.008 | 0.006 | 0.062 | -0.206 | 0.190 |
| **DPT immunization** | 0.026 | 0.005 | 0.047 | 0.011 | 0.105 | -0.356 | 0.240 |
| **economic freedom** | 0.204 | 0.187 | 0.221 | 0.009 | 0.086 | -0.014 | 0.442 |
| education | -0.012 | -0.029 | 0.005 | 0.009 | 0.085 | -0.240 | 0.205 |
| **excess mortality2** | -0.110 | -0.126 | -0.093 | 0.008 | 0.083 | -0.332 | 0.159 |
| **Facebook audience** | 0.068 | 0.047 | 0.088 | 0.010 | 0.101 | -0.340 | 0.317 |
| **female labor** | 0.093 | 0.077 | 0.109 | 0.008 | 0.080 | -0.145 | 0.313 |
| **female pop** | 0.246 | 0.221 | 0.271 | 0.013 | 0.125 | -0.017 | 0.646 |
| GDP | -0.010 | -0.040 | 0.021 | 0.015 | 0.153 | -0.512 | 0.261 |
| **gini** | -0.073 | -0.096 | -0.049 | 0.012 | 0.116 | -0.455 | 0.227 |
| **GRInon-vaccinated2** | 0.209 | 0.188 | 0.230 | 0.011 | 0.106 | 0.050 | 0.460 |
| **health and social employment** | -0.053 | -0.074 | -0.032 | 0.011 | 0.105 | -0.475 | 0.184 |
| **health expenditure** | 0.078 | 0.059 | 0.097 | 0.009 | 0.095 | -0.083 | 0.313 |
| hospital density | -0.017 | -0.042 | 0.009 | 0.013 | 0.129 | -0.429 | 0.294 |
| hospital beds | 0.009 | -0.006 | 0.025 | 0.008 | 0.077 | -0.143 | 0.354 |
| hospital employment | 0.005 | -0.017 | 0.028 | 0.011 | 0.114 | -0.345 | 0.291 |
| ICU occupancy2 | 0.000 | -0.020 | 0.021 | 0.010 | 0.102 | -0.280 | 0.273 |
| **individualism** | 0.159 | 0.138 | 0.179 | 0.010 | 0.102 | -0.302 | 0.408 |
| **indulgence** | -0.044 | -0.061 | -0.026 | 0.009 | 0.088 | -0.378 | 0.230 |
| **long-term care residents** | -0.050 | -0.063 | -0.036 | 0.007 | 0.069 | -0.228 | 0.159 |
| **long-term orientation** | -0.070 | -0.092 | -0.049 | 0.011 | 0.108 | -0.431 | 0.139 |
| **mandatory vaccination2** | 0.040 | 0.021 | 0.060 | 0.010 | 0.099 | -0.174 | 0.309 |
| **masculinity** | 0.012 | 0.000 | 0.024 | 0.006 | 0.058 | -0.129 | 0.169 |
| **MCV1 immunization** | 0.053 | 0.032 | 0.074 | 0.011 | 0.106 | -0.401 | 0.374 |
| **MDs density** | -0.038 | -0.062 | -0.013 | 0.012 | 0.125 | -0.425 | 0.208 |
| **nurses and midwives density** | 0.039 | 0.018 | 0.059 | 0.010 | 0.104 | -0.147 | 0.428 |
| **power distance** | -0.200 | -0.216 | -0.184 | 0.008 | 0.080 | -0.419 | -0.015 |
| **pharmacists density** | 0.040 | 0.013 | 0.066 | 0.013 | 0.134 | -0.316 | 0.325 |
| **population density** | 0.033 | 0.025 | 0.042 | 0.004 | 0.043 | -0.024 | 0.219 |
| **population growth** | 0.105 | 0.079 | 0.132 | 0.013 | 0.133 | -0.148 | 0.662 |
| **population size** | -0.059 | -0.072 | -0.047 | 0.006 | 0.063 | -0.212 | 0.081 |
| **population 15-64yrs** | -0.039 | -0.064 | -0.014 | 0.013 | 0.126 | -0.369 | 0.234 |
| **population 65+** | 0.047 | 0.037 | 0.058 | 0.005 | 0.053 | -0.055 | 0.249 |
| poverty | 0.000 | -0.020 | 0.020 | 0.010 | 0.100 | -0.225 | 0.261 |
| **psychiatrists density** | -0.072 | -0.091 | -0.054 | 0.009 | 0.094 | -0.277 | 0.180 |
| political stability and lack of violence/terrorism | 0.015 | -0.002 | 0.031 | 0.008 | 0.084 | -0.292 | 0.156 |
| R&D expenditure | 0.001 | -0.015 | 0.016 | 0.008 | 0.077 | -0.242 | 0.204 |
| **right-wing in government** | -0.139 | -0.154 | -0.123 | 0.008 | 0.077 | -0.326 | 0.086 |
| **smoking prevalence** | -0.052 | -0.073 | -0.031 | 0.011 | 0.106 | -0.311 | 0.221 |
| **social expenditure** | 0.076 | 0.053 | 0.098 | 0.011 | 0.113 | -0.248 | 0.371 |
| **surgeons density** | 0.056 | 0.042 | 0.071 | 0.007 | 0.074 | -0.118 | 0.215 |
| **total deaths2** | -0.092 | -0.110 | -0.075 | 0.009 | 0.088 | -0.305 | 0.133 |
| **total healthcare coverage** | 0.051 | 0.032 | 0.069 | 0.009 | 0.093 | -0.229 | 0.333 |
| **Twitter audience** | 0.052 | 0.036 | 0.067 | 0.008 | 0.078 | -0.133 | 0.283 |
| **uncertainty avoidance** | 0.170 | 0.149 | 0.190 | 0.010 | 0.105 | -0.108 | 0.522 |
| **unemployment rate** | 0.037 | 0.020 | 0.054 | 0.009 | 0.086 | -0.222 | 0.262 |
| **urban population** | 0.119 | 0.106 | 0.132 | 0.007 | 0.066 | -0.019 | 0.281 |

*Table B6. Partial least squares regression (PLSR) pooled estimates for Y3 in EU countries (covariates in bold considered to have statistically significant effects due to 95% confidence interval not containing 0; covariates listed in alphabetical order)*

| **covariate** | **mean** | **95% confidence interval lower boundary** | **95% confidence interval upper boundary** | **standard error** | **standard deviation** | **minimum value** | **maximum value** |
| --- | --- | --- | --- | --- | --- | --- | --- |
| alcohol consumption | -0.020 | -0.045 | 0.006 | 0.013 | 0.129 | -0.399 | 0.302 |
| **area** | 0.027 | 0.017 | 0.037 | 0.005 | 0.050 | -0.140 | 0.137 |
| **birth rate** | 0.049 | 0.027 | 0.071 | 0.011 | 0.111 | -0.118 | 0.478 |
| **corruption perception** | -0.038 | -0.056 | -0.019 | 0.009 | 0.091 | -0.327 | 0.123 |
| **DALYs** | -0.029 | -0.047 | -0.011 | 0.009 | 0.090 | -0.253 | 0.172 |
| democracy | -0.021 | -0.047 | 0.004 | 0.013 | 0.127 | -0.333 | 0.374 |
| **difference in GRI3** | -0.044 | -0.064 | -0.024 | 0.010 | 0.103 | -0.332 | 0.182 |
| DPT immunization | 0.002 | -0.033 | 0.038 | 0.018 | 0.181 | -0.667 | 0.325 |
| **economic freedom** | 0.070 | 0.044 | 0.096 | 0.013 | 0.130 | -0.202 | 0.487 |
| **education** | -0.141 | -0.169 | -0.112 | 0.014 | 0.144 | -0.743 | 0.194 |
| **excess mortality3** | -0.194 | -0.216 | -0.171 | 0.011 | 0.115 | -0.558 | 0.260 |
| **Facebook audience** | 0.081 | 0.056 | 0.105 | 0.012 | 0.122 | -0.179 | 0.440 |
| **female labor** | -0.088 | -0.115 | -0.061 | 0.014 | 0.138 | -0.531 | 0.167 |
| **female pop** | 0.080 | 0.053 | 0.108 | 0.014 | 0.138 | -0.258 | 0.473 |
| **GDP** | 0.105 | 0.081 | 0.128 | 0.012 | 0.117 | -0.251 | 0.370 |
| gini | 0.002 | -0.028 | 0.033 | 0.015 | 0.152 | -0.449 | 0.324 |
| **GRInon-vaccinated3** | 0.112 | 0.088 | 0.137 | 0.012 | 0.124 | -0.205 | 0.394 |
| **health and social employment** | 0.108 | 0.076 | 0.140 | 0.016 | 0.163 | -0.266 | 0.632 |
| **health expenditure** | -0.021 | -0.041 | -0.001 | 0.010 | 0.099 | -0.289 | 0.207 |
| **hospital density** | -0.048 | -0.078 | -0.017 | 0.015 | 0.153 | -0.718 | 0.353 |
| hospital beds | 0.011 | -0.014 | 0.037 | 0.013 | 0.127 | -0.202 | 0.399 |
| **hospital employment** | 0.061 | 0.025 | 0.096 | 0.018 | 0.178 | -0.343 | 0.611 |
| **ICU occupancy3** | 0.083 | 0.056 | 0.111 | 0.014 | 0.140 | -0.307 | 0.461 |
| individualism | -0.009 | -0.035 | 0.016 | 0.013 | 0.129 | -0.549 | 0.397 |
| indulgence | 0.012 | -0.009 | 0.032 | 0.010 | 0.103 | -0.215 | 0.398 |
| **long-term care residents** | -0.059 | -0.081 | -0.038 | 0.011 | 0.109 | -0.431 | 0.183 |
| **long-term orientation** | -0.054 | -0.080 | -0.028 | 0.013 | 0.131 | -0.364 | 0.392 |
| **mandatory vaccination3** | 0.032 | 0.005 | 0.059 | 0.013 | 0.135 | -0.284 | 0.400 |
| **masculinity** | 0.092 | 0.074 | 0.109 | 0.009 | 0.089 | -0.153 | 0.314 |
| **MCV1 immunization** | -0.088 | -0.111 | -0.064 | 0.012 | 0.119 | -0.437 | 0.324 |
| MDs density | -0.003 | -0.033 | 0.027 | 0.015 | 0.150 | -0.418 | 0.361 |
| **nurses and midwives density** | 0.129 | 0.099 | 0.158 | 0.015 | 0.147 | -0.206 | 0.650 |
| **power distance** | -0.126 | -0.153 | -0.098 | 0.014 | 0.140 | -0.378 | 0.459 |
| **pharmacists density** | 0.038 | 0.003 | 0.072 | 0.017 | 0.174 | -0.528 | 0.503 |
| **population density** | -0.015 | -0.023 | -0.008 | 0.004 | 0.038 | -0.116 | 0.057 |
| **population growth** | -0.213 | -0.255 | -0.171 | 0.021 | 0.212 | -0.718 | 0.374 |
| **population size** | 0.065 | 0.044 | 0.085 | 0.010 | 0.102 | -0.418 | 0.297 |
| **population 15-64yrs** | -0.081 | -0.104 | -0.058 | 0.011 | 0.114 | -0.374 | 0.204 |
| population 65+ | 0.001 | -0.014 | 0.015 | 0.007 | 0.072 | -0.226 | 0.198 |
| **poverty** | 0.040 | 0.007 | 0.073 | 0.017 | 0.168 | -0.193 | 0.591 |
| psychiatrists density | 0.023 | -0.014 | 0.060 | 0.019 | 0.187 | -0.775 | 0.589 |
| political stability and lack of violence/terrorism | -0.007 | -0.024 | 0.009 | 0.008 | 0.084 | -0.283 | 0.324 |
| **R&D expenditure** | 0.119 | 0.097 | 0.142 | 0.011 | 0.114 | -0.220 | 0.399 |
| **right-wing in government** | -0.114 | -0.143 | -0.085 | 0.015 | 0.145 | -0.561 | 0.431 |
| **smoking prevalence** | -0.050 | -0.081 | -0.020 | 0.015 | 0.153 | -0.614 | 0.336 |
| **social expenditure** | 0.037 | 0.011 | 0.064 | 0.013 | 0.134 | -0.306 | 0.369 |
| **surgeons density** | 0.079 | 0.053 | 0.104 | 0.013 | 0.130 | -0.244 | 0.498 |
| **total deaths3** | -0.052 | -0.079 | -0.025 | 0.014 | 0.136 | -0.555 | 0.360 |
| total healthcare coverage | 0.016 | -0.008 | 0.039 | 0.012 | 0.118 | -0.262 | 0.434 |
| **Twitter audience** | 0.026 | 0.006 | 0.046 | 0.010 | 0.101 | -0.253 | 0.375 |
| **uncertainty avoidance** | 0.167 | 0.141 | 0.193 | 0.013 | 0.132 | -0.142 | 0.493 |
| unemployment rate | 0.008 | -0.016 | 0.033 | 0.012 | 0.124 | -0.321 | 0.512 |
| **urban population** | 0.163 | 0.145 | 0.181 | 0.009 | 0.090 | -0.029 | 0.528 |

*Table B7. Partial least squares regression (PLSR) pooled estimates for Y1 in OECD countries (covariates in bold considered to have statistically significant effects due to 95% confidence interval not containing 0; covariates listed in alphabetical order)*

| **covariate** | **mean** | **95% confidence interval lower boundary** | **95% confidence interval upper boundary** | **standard error** | **standard deviation** | **minimum value** | **maximum value** |
| --- | --- | --- | --- | --- | --- | --- | --- |
| **alcohol consumption** | -0.073 | -0.099 | -0.048 | 0.013 | 0.129 | -0.420 | 0.180 |
| **area** | 0.120 | 0.094 | 0.146 | 0.013 | 0.131 | -0.283 | 0.437 |
| **birth rate** | -0.176 | -0.197 | -0.156 | 0.010 | 0.105 | -0.465 | 0.081 |
| **corruption perception** | 0.320 | 0.292 | 0.347 | 0.014 | 0.138 | -0.033 | 0.594 |
| DALYs | -0.008 | -0.030 | 0.014 | 0.011 | 0.110 | -0.329 | 0.247 |
| democracy | 0.022 | -0.010 | 0.055 | 0.017 | 0.166 | -0.353 | 0.355 |
| **difference in GRI1** | -0.047 | -0.068 | -0.025 | 0.011 | 0.109 | -0.325 | 0.208 |
| **DPT immunization** | -0.351 | -0.374 | -0.328 | 0.012 | 0.117 | -0.613 | -0.047 |
| **economic freedom** | -0.262 | -0.289 | -0.235 | 0.014 | 0.138 | -0.748 | 0.125 |
| education | -0.027 | -0.060 | 0.006 | 0.017 | 0.166 | -0.416 | 0.318 |
| excess mortality1 | -0.004 | -0.032 | 0.024 | 0.014 | 0.140 | -0.319 | 0.347 |
| **Facebook audience** | 0.395 | 0.365 | 0.425 | 0.015 | 0.153 | -0.057 | 0.695 |
| **female labor** | -0.085 | -0.116 | -0.053 | 0.016 | 0.158 | -0.395 | 0.303 |
| **female pop** | 0.260 | 0.232 | 0.287 | 0.014 | 0.138 | -0.099 | 0.557 |
| GDP | 0.001 | -0.024 | 0.027 | 0.013 | 0.129 | -0.404 | 0.360 |
| **gini** | -0.105 | -0.129 | -0.080 | 0.012 | 0.124 | -0.383 | 0.230 |
| **GRInon-vaccinated1** | 0.403 | 0.386 | 0.421 | 0.009 | 0.087 | 0.191 | 0.587 |
| health and social employment | 0.077 | 0.039 | 0.114 | 0.019 | 0.190 | -0.345 | 0.469 |
| **health expenditure** | -0.098 | -0.124 | -0.072 | 0.013 | 0.130 | -0.404 | 0.182 |
| **hospital density** | -0.174 | -0.201 | -0.147 | 0.014 | 0.137 | -0.610 | 0.103 |
| **hospital beds** | -0.156 | -0.188 | -0.124 | 0.016 | 0.161 | -0.497 | 0.247 |
| **hospital employment** | 0.056 | 0.021 | 0.090 | 0.017 | 0.175 | -0.419 | 0.436 |
| **ICU occupancy1** | -0.075 | -0.102 | -0.049 | 0.013 | 0.133 | -0.362 | 0.256 |
| **individualism** | -0.091 | -0.120 | -0.062 | 0.015 | 0.147 | -0.528 | 0.183 |
| **indulgence** | -0.394 | -0.420 | -0.367 | 0.013 | 0.132 | -0.693 | 0.021 |
| **long-term care residents** | -0.074 | -0.096 | -0.052 | 0.011 | 0.111 | -0.353 | 0.190 |
| **long-term orientation** | -0.056 | -0.078 | -0.035 | 0.011 | 0.109 | -0.293 | 0.142 |
| **mandatory vaccination1** | -0.387 | -0.416 | -0.359 | 0.014 | 0.144 | -0.869 | -0.078 |
| masculinity | -0.008 | -0.024 | 0.009 | 0.008 | 0.083 | -0.258 | 0.147 |
| **MCV1 immunization** | 0.246 | 0.221 | 0.271 | 0.013 | 0.126 | -0.108 | 0.629 |
| **MDs density** | -0.064 | -0.097 | -0.032 | 0.016 | 0.162 | -0.375 | 0.380 |
| **nurses and midwives density** | -0.033 | -0.063 | -0.004 | 0.015 | 0.147 | -0.371 | 0.295 |
| **power distance** | -0.130 | -0.161 | -0.098 | 0.016 | 0.158 | -0.481 | 0.359 |
| **pharmacists density** | 0.042 | 0.019 | 0.065 | 0.012 | 0.116 | -0.244 | 0.286 |
| **population density** | -0.237 | -0.251 | -0.222 | 0.007 | 0.074 | -0.443 | -0.068 |
| **population growth** | 0.059 | 0.040 | 0.079 | 0.010 | 0.098 | -0.189 | 0.385 |
| **population size** | -0.092 | -0.114 | -0.069 | 0.011 | 0.113 | -0.399 | 0.189 |
| **population 15-64yrs** | 0.169 | 0.147 | 0.190 | 0.011 | 0.110 | -0.070 | 0.408 |
| **population 65+** | 0.156 | 0.137 | 0.174 | 0.009 | 0.093 | -0.089 | 0.374 |
| **poverty** | -0.047 | -0.088 | -0.006 | 0.021 | 0.208 | -0.402 | 0.413 |
| **psychiatrists density** | 0.311 | 0.285 | 0.338 | 0.013 | 0.135 | 0.010 | 0.667 |
| **political stability and lack of violence/terrorism** | -0.146 | -0.173 | -0.119 | 0.014 | 0.137 | -0.413 | 0.266 |
| **R&D expenditure** | 0.278 | 0.255 | 0.301 | 0.012 | 0.116 | -0.021 | 0.624 |
| **right-wing in government** | -0.074 | -0.092 | -0.057 | 0.009 | 0.088 | -0.285 | 0.136 |
| smoking prevalence | -0.025 | -0.057 | 0.007 | 0.016 | 0.161 | -0.411 | 0.357 |
| social expenditure | 0.006 | -0.017 | 0.029 | 0.011 | 0.115 | -0.361 | 0.324 |
| surgeons density | -0.005 | -0.029 | 0.019 | 0.012 | 0.122 | -0.276 | 0.325 |
| **total deaths1** | 0.333 | 0.310 | 0.355 | 0.011 | 0.114 | 0.062 | 0.603 |
| **total healthcare coverage** | -0.071 | -0.096 | -0.046 | 0.013 | 0.128 | -0.390 | 0.208 |
| **Twitter audience** | 0.276 | 0.253 | 0.299 | 0.011 | 0.115 | -0.088 | 0.586 |
| **uncertainty avoidance** | -0.040 | -0.070 | -0.011 | 0.015 | 0.150 | -0.371 | 0.300 |
| **unemployment rate** | -0.125 | -0.151 | -0.099 | 0.013 | 0.129 | -0.404 | 0.123 |
| **urban population** | 0.316 | 0.293 | 0.339 | 0.012 | 0.116 | 0.033 | 0.542 |

*Table B8. Partial least squares regression (PLSR) pooled estimates for Y2 in OECD countries (covariates in bold considered to have statistically significant effects due to 95% confidence interval not containing 0; covariates listed in alphabetical order)*

| **covariate** | **mean** | **95% confidence interval lower boundary** | **95% confidence interval upper boundary** | **standard error** | **standard deviation** | **minimum value** | **maximum value** |
| --- | --- | --- | --- | --- | --- | --- | --- |
| **alcohol consumption** | 0.138 | 0.122 | 0.155 | 0.008 | 0.081 | -0.076 | 0.356 |
| **area** | -0.026 | -0.046 | -0.006 | 0.010 | 0.101 | -0.342 | 0.183 |
| **birth rate** | -0.281 | -0.299 | -0.263 | 0.009 | 0.091 | -0.560 | -0.093 |
| **corruption perception** | 0.095 | 0.070 | 0.120 | 0.013 | 0.127 | -0.416 | 0.420 |
| **DALYs** | -0.175 | -0.194 | -0.157 | 0.009 | 0.093 | -0.511 | 0.021 |
| democracy | -0.022 | -0.044 | 0.001 | 0.011 | 0.113 | -0.364 | 0.266 |
| **difference in GRI2** | 0.084 | 0.069 | 0.099 | 0.008 | 0.077 | -0.131 | 0.241 |
| **DPT immunization** | -0.093 | -0.115 | -0.072 | 0.011 | 0.108 | -0.504 | 0.177 |
| **economic freedom** | 0.247 | 0.217 | 0.277 | 0.015 | 0.150 | -0.028 | 0.696 |
| education | -0.005 | -0.028 | 0.019 | 0.012 | 0.118 | -0.330 | 0.296 |
| **excess mortality2** | -0.057 | -0.082 | -0.032 | 0.013 | 0.126 | -0.332 | 0.216 |
| **Facebook audience** | 0.323 | 0.301 | 0.346 | 0.011 | 0.113 | -0.015 | 0.704 |
| **female labor** | -0.220 | -0.241 | -0.199 | 0.010 | 0.105 | -0.533 | 0.013 |
| **female pop** | 0.345 | 0.324 | 0.366 | 0.011 | 0.107 | 0.152 | 0.721 |
| **GDP** | 0.060 | 0.025 | 0.094 | 0.017 | 0.173 | -0.377 | 0.482 |
| **gini** | 0.072 | 0.042 | 0.103 | 0.015 | 0.154 | -0.396 | 0.389 |
| **GRInon-vaccinated2** | 0.089 | 0.070 | 0.108 | 0.010 | 0.098 | -0.149 | 0.332 |
| **health and social employment** | -0.028 | -0.048 | -0.008 | 0.010 | 0.100 | -0.377 | 0.214 |
| **health expenditure** | 0.139 | 0.112 | 0.165 | 0.013 | 0.134 | -0.081 | 0.594 |
| hospital density | -0.010 | -0.034 | 0.014 | 0.012 | 0.121 | -0.346 | 0.334 |
| **hospital beds** | 0.053 | 0.033 | 0.074 | 0.010 | 0.104 | -0.196 | 0.403 |
| **hospital employment** | -0.030 | -0.051 | -0.009 | 0.011 | 0.107 | -0.279 | 0.243 |
| ICU occupancy2 | -0.013 | -0.034 | 0.007 | 0.010 | 0.103 | -0.319 | 0.177 |
| **individualism** | -0.114 | -0.138 | -0.090 | 0.012 | 0.123 | -0.471 | 0.290 |
| **indulgence** | -0.082 | -0.105 | -0.059 | 0.011 | 0.114 | -0.433 | 0.136 |
| **long-term care residents** | -0.084 | -0.098 | -0.069 | 0.007 | 0.072 | -0.247 | 0.175 |
| **long-term orientation** | -0.150 | -0.172 | -0.128 | 0.011 | 0.111 | -0.460 | 0.049 |
| **mandatory vaccination2** | 0.030 | 0.010 | 0.051 | 0.010 | 0.105 | -0.279 | 0.248 |
| **masculinity** | -0.060 | -0.072 | -0.048 | 0.006 | 0.059 | -0.309 | 0.056 |
| **MCV1 immunization** | 0.166 | 0.148 | 0.184 | 0.009 | 0.090 | -0.086 | 0.381 |
| **MDs density** | -0.046 | -0.079 | -0.013 | 0.017 | 0.166 | -0.505 | 0.269 |
| **nurses and midwives density** | 0.094 | 0.069 | 0.119 | 0.013 | 0.126 | -0.160 | 0.519 |
| **power distance** | -0.106 | -0.125 | -0.088 | 0.009 | 0.094 | -0.354 | 0.135 |
| **pharmacists density** | 0.109 | 0.082 | 0.135 | 0.013 | 0.134 | -0.177 | 0.435 |
| **population density** | -0.200 | -0.212 | -0.189 | 0.006 | 0.059 | -0.386 | -0.072 |
| **population growth** | -0.114 | -0.137 | -0.091 | 0.012 | 0.116 | -0.392 | 0.095 |
| **population size** | -0.034 | -0.051 | -0.016 | 0.009 | 0.087 | -0.252 | 0.167 |
| **population 15-64yrs** | 0.100 | 0.082 | 0.117 | 0.009 | 0.087 | -0.126 | 0.332 |
| **population 65+** | 0.064 | 0.050 | 0.077 | 0.007 | 0.068 | -0.126 | 0.252 |
| **poverty** | -0.050 | -0.074 | -0.027 | 0.012 | 0.117 | -0.262 | 0.205 |
| **psychiatrists density** | -0.049 | -0.069 | -0.029 | 0.010 | 0.100 | -0.268 | 0.288 |
| **political stability and lack of violence/terrorism** | -0.126 | -0.153 | -0.098 | 0.014 | 0.139 | -0.543 | 0.109 |
| **R&D expenditure** | 0.067 | 0.047 | 0.088 | 0.010 | 0.103 | -0.300 | 0.283 |
| right-wing in government | 0.000 | -0.013 | 0.014 | 0.007 | 0.067 | -0.175 | 0.197 |
| **smoking prevalence** | -0.037 | -0.062 | -0.013 | 0.013 | 0.125 | -0.313 | 0.299 |
| **social expenditure** | -0.046 | -0.071 | -0.021 | 0.013 | 0.126 | -0.467 | 0.155 |
| **surgeons density** | 0.028 | 0.006 | 0.051 | 0.011 | 0.113 | -0.304 | 0.262 |
| **total deaths2** | -0.090 | -0.112 | -0.068 | 0.011 | 0.111 | -0.464 | 0.136 |
| **total healthcare coverage** | 0.153 | 0.132 | 0.175 | 0.011 | 0.109 | -0.120 | 0.427 |
| **Twitter audience** | 0.129 | 0.113 | 0.145 | 0.008 | 0.080 | -0.083 | 0.314 |
| **uncertainty avoidance** | 0.078 | 0.055 | 0.101 | 0.012 | 0.116 | -0.183 | 0.337 |
| **unemployment rate** | -0.079 | -0.099 | -0.059 | 0.010 | 0.101 | -0.309 | 0.185 |
| **urban population** | 0.258 | 0.243 | 0.272 | 0.007 | 0.072 | 0.022 | 0.416 |

*Table B9. Partial least squares regression (PLSR) pooled estimates for Y3 in OECD countries (covariates in bold considered to have statistically significant effects due to 95% confidence interval not containing 0; covariates listed in alphabetical order)*

| **covariate** | **mean** | **95% confidence interval lower boundary** | **95% confidence interval upper boundary** | **standard error** | **standard deviation** | **minimum value** | **maximum value** |
| --- | --- | --- | --- | --- | --- | --- | --- |
| **alcohol consumption** | 0.085 | 0.051 | 0.120 | 0.018 | 0.175 | -0.419 | 0.714 |
| **area** | -0.189 | -0.227 | -0.151 | 0.019 | 0.192 | -0.611 | 0.346 |
| **birth rate** | -0.379 | -0.411 | -0.347 | 0.016 | 0.160 | -0.888 | -0.084 |
| **corruption perception** | 0.313 | 0.277 | 0.349 | 0.018 | 0.182 | -0.107 | 0.882 |
| **DALYs** | -0.140 | -0.174 | -0.105 | 0.017 | 0.173 | -0.611 | 0.260 |
| democracy | -0.031 | -0.074 | 0.011 | 0.021 | 0.212 | -0.622 | 0.537 |
| **difference in GRI3** | 0.066 | 0.028 | 0.104 | 0.019 | 0.192 | -0.424 | 0.793 |
| **DPT immunization** | -0.098 | -0.138 | -0.059 | 0.020 | 0.199 | -0.834 | 0.306 |
| **economic freedom** | -0.079 | -0.132 | -0.026 | 0.027 | 0.268 | -0.867 | 0.506 |
| **education** | -0.176 | -0.219 | -0.134 | 0.021 | 0.214 | -0.788 | 0.329 |
| **excess mortality3** | -0.345 | -0.395 | -0.295 | 0.025 | 0.253 | -0.957 | 0.713 |
| **Facebook audience** | 0.146 | 0.102 | 0.189 | 0.022 | 0.221 | -0.400 | 0.887 |
| **female labor** | -0.203 | -0.244 | -0.161 | 0.021 | 0.210 | -0.763 | 0.228 |
| **female pop** | 0.138 | 0.099 | 0.176 | 0.020 | 0.196 | -0.866 | 0.617 |
| **GDP** | -0.210 | -0.249 | -0.170 | 0.020 | 0.200 | -0.774 | 0.342 |
| **gini** | 0.469 | 0.435 | 0.503 | 0.017 | 0.171 | 0.069 | 0.962 |
| **GRInon-vaccinated3** | 0.271 | 0.240 | 0.302 | 0.016 | 0.156 | -0.145 | 0.583 |
| **health and social employment** | 0.337 | 0.285 | 0.388 | 0.026 | 0.260 | -0.269 | 0.940 |
| health expenditure | 0.018 | -0.013 | 0.049 | 0.016 | 0.159 | -0.406 | 0.440 |
| **hospital density** | -0.216 | -0.256 | -0.177 | 0.020 | 0.198 | -0.676 | 0.411 |
| **hospital beds** | 0.213 | 0.177 | 0.249 | 0.018 | 0.181 | -0.208 | 0.639 |
| **hospital employment** | -0.113 | -0.150 | -0.075 | 0.019 | 0.189 | -0.535 | 0.387 |
| ICU occupancy3 | -0.008 | -0.049 | 0.033 | 0.021 | 0.206 | -0.458 | 0.524 |
| **individualism** | -0.087 | -0.126 | -0.048 | 0.020 | 0.199 | -0.767 | 0.521 |
| **indulgence** | -0.171 | -0.213 | -0.129 | 0.021 | 0.211 | -0.838 | 0.359 |
| **long-term care residents** | -0.084 | -0.117 | -0.052 | 0.016 | 0.165 | -0.508 | 0.330 |
| **long-term orientation** | -0.240 | -0.276 | -0.205 | 0.018 | 0.179 | -0.604 | 0.150 |
| mandatory vaccination3 | -0.003 | -0.026 | 0.020 | 0.012 | 0.116 | -0.334 | 0.250 |
| **masculinity** | -0.047 | -0.073 | -0.021 | 0.013 | 0.133 | -0.347 | 0.434 |
| **MCV1 immunization** | -0.205 | -0.244 | -0.166 | 0.020 | 0.197 | -0.891 | 0.227 |
| MDs density | -0.006 | -0.050 | 0.037 | 0.022 | 0.219 | -0.482 | 0.454 |
| **nurses and midwives density** | 0.143 | 0.103 | 0.182 | 0.020 | 0.199 | -0.548 | 0.576 |
| power distance | 0.006 | -0.029 | 0.041 | 0.018 | 0.175 | -0.399 | 0.511 |
| **pharmacists density** | 0.231 | 0.193 | 0.269 | 0.019 | 0.193 | -0.389 | 0.674 |
| **population density** | 0.068 | 0.053 | 0.084 | 0.008 | 0.080 | -0.142 | 0.240 |
| **population growth** | -0.075 | -0.101 | -0.049 | 0.013 | 0.129 | -0.339 | 0.298 |
| **population size** | -0.268 | -0.295 | -0.240 | 0.014 | 0.138 | -0.584 | 0.108 |
| **population 15-64yrs** | 0.062 | 0.029 | 0.095 | 0.016 | 0.164 | -0.474 | 0.532 |
| **population 65+** | 0.117 | 0.081 | 0.154 | 0.019 | 0.185 | -0.282 | 0.861 |
| **poverty** | -0.188 | -0.241 | -0.136 | 0.026 | 0.264 | -0.757 | 0.447 |
| **psychiatrists density** | 0.052 | 0.020 | 0.085 | 0.016 | 0.163 | -0.518 | 0.407 |
| **political stability and lack of violence/terrorism** | -0.289 | -0.345 | -0.233 | 0.028 | 0.282 | -1.027 | 0.463 |
| R&D expenditure | 0.014 | -0.025 | 0.053 | 0.020 | 0.198 | -0.455 | 0.622 |
| right-wing in government | 0.006 | -0.022 | 0.033 | 0.014 | 0.140 | -0.382 | 0.309 |
| **smoking prevalence** | -0.089 | -0.142 | -0.036 | 0.027 | 0.266 | -0.766 | 0.517 |
| **social expenditure** | -0.188 | -0.225 | -0.151 | 0.019 | 0.188 | -0.841 | 0.191 |
| **surgeons density** | 0.113 | 0.079 | 0.147 | 0.017 | 0.169 | -0.288 | 0.531 |
| **total deaths3** | 0.160 | 0.129 | 0.191 | 0.015 | 0.155 | -0.284 | 0.634 |
| **total healthcare coverage** | -0.123 | -0.159 | -0.087 | 0.018 | 0.184 | -0.898 | 0.295 |
| **Twitter audience** | 0.043 | 0.003 | 0.082 | 0.020 | 0.200 | -0.566 | 0.742 |
| **uncertainty avoidance** | -0.053 | -0.092 | -0.014 | 0.020 | 0.195 | -0.634 | 0.351 |
| **unemployment rate** | -0.185 | -0.220 | -0.151 | 0.017 | 0.175 | -0.626 | 0.207 |
| **urban population** | 0.398 | 0.362 | 0.434 | 0.018 | 0.182 | -0.233 | 1.009 |
